# Desperately Seeking Status: How Desires for, and Perceived Attainment of, Status and Inclusion Relate to Grandiose and Vulnerable Narcissism

**DOI:** 10.1177/01461672211021189

**Published:** 2021-06-11

**Authors:** Nikhila Mahadevan, Christian Jordan

**Affiliations:** 1 University of Essex, Colchester, Essex, UK; 2 Wilfrid Laurier University, Waterloo, Ontario, Canada

**Keywords:** narcissism, grandiose narcissism, vulnerable narcissism, social status, social inclusion

## Abstract

The desire for social status is theorized as being central to narcissism; however, research to date has focused exclusively on grandiose narcissism. We examined how desires for, and perceived attainment of, status and inclusion relate to grandiose narcissism, vulnerable narcissism, and three-factor models of narcissism. Two studies (total *N* = 676) found that all expressions of narcissism relate to a stronger desire for status. Within three-factor models, this relation was not due solely to variance shared by grandiose and vulnerable narcissism, but also to phenotype-specific components. Grandiose narcissism was also strongly associated with perceived attainment of status, but not desire for or perceived attainment of inclusion, whereas vulnerable narcissism was strongly associated with desire for inclusion, but not perceived attainment of status or inclusion. Three-factor models of narcissism revealed comparable results. The findings delineate the social and motivational profiles of different expressions of narcissism, helping to illuminate narcissism’s fundamental character.

Narcissism is a complex constellation of diverse personality features (Krizan & Herlache, 2018; Miller et al., 2017; Morf & Rhodewalt, 2001). Several lines of research converge on the idea that a strong desire for social status is central to narcissism and may, in fact, create coherence among narcissistic personality characteristics (Grapsas et al., 2020; Mahadevan et al., 2016; Zeigler-Hill et al., 2019). This possibility is consistent with the fact that narcissistic individuals are hypercompetitive (Luchner et al., 2011), strive to display superiority and dominance (Brown & Zeigler-Hill, 2004), and push to achieve leadership positions in groups (Nevicka, 2018). Grandiose narcissism relates consistently to status aspirations (even when other social motives are controlled) and narcissistic individuals are notably sensitive to threats to social status (Mahadevan et al., 2020; Zeigler-Hill et al., 2019). A strong desire for status may thus be a unifying motive underlying narcissistic personality features. Existing theory and research in this area, however, has focused exclusively on grandiose narcissism and neglected vulnerable narcissism. The primary purpose of the present research is to test whether a desire for status also characterizes vulnerable narcissism. If so, a common desire for status may further unify and give coherence to these disparate expressions of narcissism.

Theory and research support a long-standing distinction between *grandiose* and *vulnerable* narcissism (Cain et al., 2008). Grandiose narcissism is bold, extraverted, and immodest, whereas vulnerable narcissism is withdrawn, neurotic, and insecure. These expressions of narcissism have frequently diverging nomological networks (Dickinson & Pincus, 2003; Miller et al., 2011). Identifying common features and motives underlying both grandiose and vulnerable narcissism can therefore inform a better understanding of the fundamental nature of narcissism.

If both grandiose and vulnerable narcissism are characterized by a strong desire for status, it is also important to further determine the nature of this common association. Recently, two lines of research—the *Narcissism Spectrum Model* (NSM; Krizan & Herlache, 2018) and the *trifurcated model of narcissism* (Weiss et al., 2019)—suggest the narcissistic personality reflects three underlying dimensions: (a) *Agentic extraversion* or *grandiosity* which characterizes grandiose narcissism specifically, (b) *narcissistic neuroticism* or *vulnerability* which characterizes vulnerable narcissism specifically, and (c) *antagonism* or *entitlement* which is common to both. These dimensions highlight the structural commonalities between grandiose and vulnerable narcissism. Additional research, however, can help specify functional commonalities between them, such as a shared desire for status. In the present research, we explore whether a strong desire for status also unifies these more specific expressions of narcissism.

Notably, if both grandiose and vulnerable narcissism relate to a desire for status, further considering a three-dimensional structure can reveal whether this common relation reflects only shared structural variance between them (i.e., antagonism or entitlement) or whether it also characterizes phenotype-specific components (i.e., agentic extraversion/grandiosity and narcissistic neuroticism/vulnerability). We expect a desire for status unifies all aspects of narcissism, including the unique aspects.

Finally, to provide a more comprehensive picture of the social and motivational profile underlying narcissism, we examine whether grandiose and vulnerable narcissism (as well as three-factor expressions) differ in how they relate to perceived attainment of status, and desire for, and perceived attainment of, social inclusion. We expect that a strong desire for status characterizes *all* expressions of narcissism, but that perceived attainment of status, desire for inclusion, and perceived attainment of inclusion differentiate expressions of narcissism.

## Status and Inclusion

*Social status* is defined as the extent to which an individual is *respected and admired* by others (Fiske, 2010). In contrast, *social inclusion* is defined as the extent to which an individual is *liked and accepted* by others (Fournier, 2009). The needs for status and inclusion are both considered to be fundamental human motives (Anderson et al., 2015; Baumeister & Leary, 1995). These motives are separate from and non-derivative of one another.

Status and inclusion share some similarities. Both are social, involve the opinions and evaluations of others, and are positively correlated (Mahadevan et al., 2019a, 2019b). Nonetheless, they are conceptually and empirically distinct (Anderson et al., 2015). A person can be high in both, low in both, or high in one but low in the other. For example, a person may have high status, but not be liked and accepted (e.g., Mr Burns), or may be well-liked and accepted, but not have high status (e.g., Homer Simpson). Whereas status denotes one’s “vertical” position in the social hierarchy, inclusion denotes one’s “horizontal” position in the social community.

Indeed, the status-inclusion distinction may be understood in terms of the overarching *agency-communion distinction*. Agency and communion constitute two basic dimensions in social cognition (“The Big Two”; Abele & Wojciszke, 2018). Several phenomena fall under this broad distinction, including masculinity-femininity, competence-warmth, dominance-agreeableness, and independence-interdependence (Gregg et al., 2017a). Status is agentic—it involves *getting ahead—*whereas inclusion is communal—it involves *getting along* (Hogan & Holland, 2003).

## Status and Inclusion: Perceived Attainment vs. Aspirations

People differ in the extent to which they possess, or see themselves as possessing, status and inclusion. Some individuals are highly respected and admired by others (i.e., have high status attainment), and others less so (i.e., have low status attainment). Likewise, some individuals are well-liked and accepted by others (i.e., have high inclusion attainment), and others less so (i.e., have low inclusion attainment). In the current studies, we focus on self-perceived attainment of status and inclusion. Although self-perceptions are imperfect indicators of attainment, they affect important decisions and behaviors. In addition, self-perceptions of status and inclusion are fairly accurate: People’s own ratings of their status and inclusion correlate positively with peer-ratings (Anderson et al., 2006; Fournier, 2009).

Higher status attainment—whether self-perceived or other-perceived—is associated with better psychological health. For example, higher status people feel less anxious (Gregg et al., 2018) and report greater life satisfaction (Anderson et al., 2012). Likewise, higher inclusion attainment—whether self-perceived or other-perceived—is associated with better psychological health. Socially included people are less angry and aggressive (Twenge et al., 2001), and experience life as more meaningful (Stillman et al., 2009). Thus, higher attainment of status and inclusion are associated with better psychological health.

Not only do people differ in the extent to which they feel they possess status or inclusion, they also differ in the extent to which they desire each (Neel et al., 2016). Although the desires for status and inclusion are both considered to be fundamental motives, there are nonetheless individual differences in the extent to which people experience these desires. Individuals typically differ in the degree to which they experience various motives, even fundamental motives (e.g., libido; Gregg & Mahadevan, 2014; Sheldon & Schüler, 2011). Whereas some individuals keenly desire the respect and admiration of others (i.e., have high status aspirations), others do not (i.e., have low status aspirations). Likewise, whereas some individuals keenly desire the liking and acceptance of others (i.e., have high inclusion aspirations), others do not (i.e., have low inclusion aspirations).

Unlike higher perceived attainment of status and inclusion, higher aspirations for status and inclusion are associated with worse psychological health. For instance, people with higher aspirations for status and inclusion report lower self-esteem and greater anxiety (Mahadevan et al., 2019b). Furthermore, people with a high need for popularity are more likely to feel depressed and to misuse alcohol and drugs (Santor et al., 2000). Thus, higher aspirations for status and inclusion appear to be associated with greater psychological vulnerability.

## The Current Research

The current research examined, for the first time, how aspirations for status and inclusion and perceived attainment of status and inclusion relate to the full spectrum of narcissistic personality features. Specifically, we examined how they relate to grandiose narcissism, vulnerable narcissism, and the three dimensions of the NSM and trifurcated model of narcissism, namely, agentic extraversion (grandiosity), self-centered antagonism (entitlement), and narcissistic neuroticism (vulnerability; Krizan & Herlache, 2018; Miller et al., 2017). Consistent with models that argue a desire for status is a unifying feature that gives coherence to the narcissistic personality (Grapsas et al., 2020; Mahadevan et al., 2016; Zeigler-Hill et al., 2019), our primary hypothesis was that all these expressions of narcissism relate positively to aspirations for status; not only grandiose and vulnerable narcissism but also their phenotype-specific components, in addition to their shared components. We hypothesized, however, that different expressions of narcissism would diverge in their perceived attainment of status, and in their aspirations for and perceived attainment of inclusion.

We hypothesized that grandiose narcissism would be associated with higher aspirations for, and higher perceived attainment of, status, but would not be associated with higher aspirations for, and higher perceived attainment of, inclusion. Several lines of theorizing and research indicate that grandiose narcissism is characterized by a preference for agency over communion (e.g., the extended agency model, Campbell & Foster, 2007). Individuals high in grandiose narcissism desire power and status, but avoid interpersonal closeness and intimacy (Foster et al., 2006). They see themselves as better-than-average on agentic traits (e.g., competence), but not communal traits (e.g., kindness; Campbell et al., 2002). They also tend to behave in dominant, but disagreeable, ways (Twenge & Campbell, 2003). Finally, recent evidence links grandiose narcissism to desire for status, even when other social motives (such as desire for affiliation) are controlled (Zeigler-Hill et al., 2019). Thus, we hypothesized that those high in grandiose narcissism would aspire for, and see themselves as having attained, high status (after controlling for inclusion aspirations and perceived inclusion attainment), whereas they would neither aspire for, nor see themselves as having attained, high inclusion (after controlling for status aspirations and perceived status attainment).

In contrast, we hypothesized that vulnerable narcissism would relate to higher aspirations for, but lower perceived attainment of, status. Like their grandiose counterparts, individuals high in vulnerable narcissism are thought to have grandiose expectations and a sense of entitlement, which may feed a desire for status (Dickinson & Pincus, 2003). However, unlike their grandiose counterparts, they do not appear to be successful at attaining status. Vulnerable narcissism is associated with low agency in the context of the interpersonal circumplex (Miller et al., 2012; Rogoza et al., 2019). It also relates to low self-esteem and submissive behavior (Back et al., 2013)—attributes linked to low status attainment (Anderson et al., 2001). It has also been theorized that narcissistic vulnerability may result when narcissistic needs or expectations go unmet (Pincus & Lukowitsky, 2010; Wright & Edershile, 2018). If so, narcissistic vulnerability should be associated with the perception that one’s expectations, such as aspirations for high status, are not fulfilled.

We also tentatively hypothesized that vulnerable narcissism would be associated with higher aspirations for, but lower perceived attainment of, inclusion. Vulnerable narcissism is associated with antagonistic and hostile responses to others—behaviors likely to result in low inclusion attainment (Miller et al., 2012). However, those high in vulnerable narcissism also report an anxious or fearful attachment style (Dickinson & Pincus, 2003), suggesting they desire to maintain close relationships, but are unable to do so. More generally, vulnerable narcissism is characterized by neuroticism and psychological vulnerability—attributes that have been linked to high aspirations for, but low attainment of, both status and inclusion (Anderson et al., 2001; Mahadevan et al., 2019b). Hence, we hypothesized that those high in vulnerable narcissism would aspire for, but not see themselves as having attained, high status (after controlling for inclusion aspirations and perceived inclusion attainment), and would aspire for, but not see themselves as having attained, high inclusion (after controlling for status aspirations and perceived status attainment).

With respect to more specific expressions of narcissism, our hypotheses for agentic extraversion and narcissistic neuroticism were identical to those for grandiose and vulnerable narcissism, respectively, because agentic extraversion is unique to grandiose narcissism and narcissistic neuroticism is unique to vulnerable narcissism. Our hypotheses for antagonism were more tentative. Recent theorizing suggests that narcissistic rivalry (which is closely related to antagonism) motivates vigilance for threats to status and may relate negatively to perceived attainment of status due to pre-emptive self-protection (Grapsas et al., 2020; Zeigler-Hill et al., 2019). If so, we expect antagonism to relate to lower perceived attainment of status, particularly relative to aspirations for status (i.e., high aspirations are not being met). Because antagonism reflects low agreeableness, we expected it to negatively or only weakly relate to aspirations for and perceived attainment of inclusion (particularly with aspirations for and perceived attainment of status controlled).

We conducted two studies. Both studies examined the links between aspirations for status and inclusion, perceived attainment of status and inclusion, and grandiose and vulnerable narcissism. Study 1 included measures identified within the NSM as best reflecting dimensions of grandiosity, vulnerability, and entitlement. Study 2 re-examined these links with alternative measures of grandiose and vulnerable narcissism, which also assess the three dimensions of narcissism described by the trifurcated model, namely, agentic extraversion, self-centered antagonism, and narcissistic neuroticism.

## Data Analytic Approach

In both studies, we focus first on zero-order correlations to test our predictions. These reflect the overall relation between expressions of narcissism and aspirations for, and perceived attainment of, status and inclusion. We then implement several kinds of control analyses. First, when testing the relations of narcissism to aspirations for status, we control aspirations for inclusion (and vice versa). This is because aspirations for status and inclusion correlate positively (e.g., Zeigler-Hill et al., 2019), reflecting a common motivation for social validation. In addition, each aspiration may also “bleed” into the other, as some individuals may, for example, view inclusion as a means to gain status (e.g., by ingratiating oneself to others in important positions, or seeking to associate with high status individuals; Campbell, 1999), or vice versa. Examining the unique associations of each aspiration, therefore, allows a clearer sense of the unique motivations of narcissistic individuals. Similarly, we control perceived attainment of inclusion when examining relations of narcissism with perceived attainment of status (and vice versa).

Second, we control aspirations for status when examining the relation of narcissism to perceptions of status. This analysis tests perceived attainment of status *relative to* aspirations for status, to give a sense of whether individuals high in narcissism feel they have attained a level of status commensurate to their aspirations. Similarly, we control aspirations for inclusion when testing perceived inclusion, to test whether individuals high in narcissism see themselves as having achieved inclusion commensurate with their aspirations.

These control analyses implemented individually require four sets of regression analyses, regressing each expression of narcissism on (a) status and inclusion aspirations, (b) perceived status and inclusion attainment, (c) status aspirations and perceived status, and (d) inclusion aspirations and perceived status. However, in both studies, we conducted these four sets of regression analyses and compared their results to those of a single set of regression analyses in which each expression of narcissism is regressed simultaneously on aspirations for status and inclusion, as well as perceived attainment of status and inclusion. The results in each case were nearly identical in both magnitude and significance. Accordingly, for simplicity, we report only this latter set of regression analyses, which implement all of the controls described above simultaneously.

In both studies, we ensured the samples exceeded 250 participants, the sample size at which correlations and regression coefficients are expected to stabilize (Schönbrodt & Perugini, 2013). However, we deliberately oversampled in anticipation of data exclusions. At no point did we stop and restart data collection; we analyzed the data only once the full reported sample had been collected. Sensitivity analyses indicated that for Study 1, the final sample size of 309 allowed detection of small-to-medium effects of ρ ≥ .18 with 90% power at α = .05 (two-tailed), and for Study 2, the final sample size of 367 allowed detection of small-to-medium effects of ρ ≥ .17 with 90% power at α = .05 (two-tailed). The data, analysis code, and materials for both studies are available at: https://osf.io/bfrkz/?view_only=5b76eb065a524b11a89c038b655f00ae.

## Study 1

Study 1 examined the associations between aspirations for status and inclusion, perceived attainment of status and inclusion, and levels of grandiose and vulnerable narcissism. We used the measures of narcissism identified by Krizan and Herlache (2018) as best representing the three underlying dimensions of the NSM: grandiosity (the Narcissistic Personality Inventory; NPI), vulnerability (the vulnerable narcissism subscale of the Pathological Narcissism Inventory; PNI), and entitlement (the Psychological Entitlement Scale; PES). Other evidence also suggests that the NPI is the most representative measure of grandiose narcissism overall, and the vulnerable narcissism subscale of the PNI is the most representative measure of vulnerable narcissism (Crowe et al., 2019). Accordingly, within this study, we refer to the NPI as a measure of grandiose narcissism, the PNI subscale as a measure of vulnerable narcissism, and the PES as a measure of entitlement.

### Method

#### Procedure

The study was hosted online on *Qualtrics*^TM^. Participants were recruited via *Amazon Mechanical Turk*^TM^ for a fee. Participants read an information sheet that described the study’s aims in general terms. They indicated their consent to take part by checking a box. Participants then completed the questionnaire measures, along with basic demographic information.

#### Participants

A total of 429 attempts were logged online for the study. Of these, we excluded 120 that appeared to be of dubious quality (Gregg et al., 2017b). Specifically, we excluded cases where participants (a) completed the study multiple times (4.9%), (b) completed the study either too quickly or too slowly (in under a third, or over thrice, the median completion time; 6.5%), (c) had too much missing data (over 10% of questionnaire items; 6.5%), (d) showed invariant responses to questionnaires containing both forward-coded and reverse-coded items (9.3%); or (e) reported that their data were not trustworthy (0.6%). The final sample consisted of 309 participants (167 men, 140 women, 1 other, and 1 unspecified) who ranged in age from 19 to 72 years (*M =* 36.86, *SD =* 11.19). Participants predominantly identified their backgrounds as White/European (71.2%), followed by Black/African/Caribbean (16.2%), Southeast Asian (4.9%), Latin American (3.2%), South Asian (1.9%), and Other (2.6%).

### Measures

#### Status aspirations

Aspirations for status were assessed using a 10-item questionnaire (Mahadevan et al., 2019b). Participants indicated the extent to which they generally desire status (e.g., “I aspire, first and foremost, to be a person of importance and distinction”; 1 = *strongly disagree*, 5 = *strongly agree*).

#### Inclusion aspirations

Aspirations for inclusion were assessed using a parallel 10-item questionnaire (Mahadevan et al., 2019b). Participants indicated the extent to which they generally desire to be included (e.g., “I desire, first and foremost, to have many friends and close relationships”; 1 = *strongly disagree*, 5 = *strongly agree*).

#### Perceived status attainment

Perceived attainment of status was assessed using an 8-item questionnaire (Huo et al., 2010; Mahadevan et al., 2019a, 2019b). Participants indicated the extent to which they feel that others generally respect and admire them (e.g., “I feel that people see me as an important person”; 1 = *strongly disagree*, 5 = *strongly agree*).

#### Perceived inclusion attainment

Perceived attainment of inclusion was assessed using a parallel 9-item questionnaire (Huo et al., 2010; Mahadevan et al., 2019a, 2019b). Participants indicated the extent to which they feel that others generally like and accept them (e.g., “I feel that people like me as a person”; 1 = *strongly disagree*, 5 = *strongly agree*).

#### Grandiose narcissism

Grandiose narcissism was assessed using the 40-item *Narcissistic Personality Inventory* (Raskin & Terry, 1988). The NPI consists of 40 pairs of statements, one non-narcissistic (e.g., “My body is nothing special”) and one narcissistic (e.g., “I like to look at my body”). Participants indicated their narcissism by choosing the statement that fit them better.

#### Psychological entitlement

Psychological entitlement was assessed using the 9-item *Psychological Entitlement Scale* (Campbell et al., 2004). Participants indicated the extent to which they generally feel entitled (e.g., “I honestly feel I’m just more deserving than others”; 1 = *strongly disagree*, 7 = *strongly agree*).

#### Vulnerable narcissism

Vulnerable narcissism was assessed using the vulnerability subscale of the 52-item *Pathological Narcissism Inventory* (Pincus et al., 2009). The PNI assesses seven aspects of pathological narcissism—Entitlement Rage, Exploitativeness, Grandiose Fantasy, Self-Sacrificing Self-Enhancement, Contingent Self-Esteem, Hiding the Self, and Devaluing. The latter three aspects assess narcissistic vulnerability and were used as the measure of vulnerable narcissism in this study (e.g., “I need others to acknowledge me [Contingent Self-Esteem]”; “It’s hard to show others the weaknesses I feel inside [Hiding the Self]”; and “Sometimes I avoid people because I’m concerned they’ll disappoint me [Devaluing]”). Participants indicated the extent to which each statement generally described them (1 = *not at all like me*, 6 = *very much like me*).^1^

### Results

Table 1 shows the descriptive statistics, internal consistencies, and zero-order correlations for all variables. All measures showed good internal consistency. Status aspirations correlated positively with inclusion aspirations. Likewise, perceived status attainment correlated positively with perceived inclusion attainment. Thus, participants who aspired to higher status also aspired to higher inclusion, and participants who perceived themselves to be higher in status also perceived themselves to be higher in inclusion. Status aspirations correlated positively with perceived status attainment. Inclusion aspirations were uncorrelated with perceived inclusion attainment.

**Table 1.** Study 1: Descriptive Statistics and Inter-Correlations for the Main Variables.

Variable	1	2	3	4	5	6	7
1. Status aspirations	1	—	—	—	—	—	—
2. Inclusion aspirations	.66***	1	—	—	—	—	—
3. Perceived status attainment	.40***	.25***	1	—	—	—	—
4. Perceived inclusion attainment	.08	.08	.72***	1	—	—	—
5. Grandiose narcissism (NPI)	.60***	.22***	.39***	.07	1	—	—
6. Entitlement (PES)	.67***	.33***	.34***	−.01	.72***	1	—
7. Vulnerable narcissism (PNI)	.58***	.55***	−.08	−.36***	.29***	.44***	1
*Mean*	3.01	2.85	3.57	3.96	9.34	3.54	14.34
*SD*	.93	1.00	.94	.83	3.16	1.58	9.44
*Cronbach’s alpha*	.90	.93	.92	.93	.93	.92	.94

*Note*. We assessed status aspirations and inclusion aspirations using two structurally validated questionnaires developed by Mahadevan et al. (2019b). We assessed perceived status attainment and perceived inclusion attainment using two structurally validated questionnaires adapted from Huo et al. (2010). We assessed grandiose narcissism using the Narcissistic Personality Inventory (Raskin & Terry, 1988) and vulnerable narcissism using the vulnerability subscale of the Pathological Narcissism Inventory (Pincus et al., 2009). We assessed psychological entitlement using the Psychological Entitlement Scale (Campbell et al., 2004).

* *p* < .05, **p < .01, ***p < .001.

#### Zero-order correlations

We began by examining the correlations between aspirations for status and inclusion, perceived attainment of status and inclusion, and expressions of narcissism (Table 1). Status aspirations correlated strongly positively with all expressions of narcissism: grandiose narcissism, entitlement, and vulnerable narcissism. Perceived status attainment correlated moderately positively with grandiose narcissism and entitlement, but was uncorrelated with vulnerable narcissism. Inclusion aspirations correlated moderately positively with grandiose narcissism and entitlement, and strongly positively with vulnerable narcissism. Notably, grandiose narcissism and entitlement were considerably more strongly related to status aspirations than inclusion aspirations (both *Z* > 5.05, *p* < .001). Vulnerable narcissism was strongly related to both. Perceived inclusion attainment was uncorrelated with grandiose narcissism and entitlement, and correlated moderately negatively with vulnerable narcissism.

#### Regression analyses

Next, we examined the unique associations of aspirations for and attainment of status and inclusion with each expression of narcissism by regressing each narcissism measure (independently) onto all four predictors (Table 2).

**Table 2.** Study 1: Standardized Regression Coefficients for Regression of Aspirations for, and Perceived Attainment of, Status and Inclusion on Narcissism.

*Variable*	Grandiose narcissism (NPI)	Entitlement (PES)	Vulnerable narcissism (PNI)
Status aspirations	.66***	.69***	.41***
Inclusion aspirations	−.28***	−.17**	.31***
Perceived status attainment	.35***	.29***	−.03
Perceived inclusion attainment	−.21***	−.26***	−.40***

*Note*. We assessed status aspirations and inclusion aspirations using two structurally validated questionnaires developed by Mahadevan et al. (2019b). We assessed perceived status attainment and perceived inclusion attainment using two structurally validated questionnaires adapted from Huo et al. (2010). We assessed grandiose narcissism using the Narcissistic Personality Inventory (Raskin & Terry, 1988) and vulnerable narcissism using the vulnerability subscale of the Pathological Narcissism Inventory (Pincus et al., 2009). We assessed psychological entitlement using the Psychological Entitlement Scale (Campbell et al., 2004).

* *p* < .05, **p < .01, ***p < .001.

Status aspirations remained strongly positively related to grandiose narcissism, *t*(304) = 10.57, *p* < .0001, entitlement, *t*(303) = 11.66, *p* < .0001, and vulnerable narcissism, *t*(304) = 7.44, *p* < .0001. In contrast, perceived status attainment remained moderately positively related to grandiose narcissism, *t*(304) = 4.90, *p* < .0001, and entitlement, *t*(303) = 4.33, *p* < .0001, but not vulnerable narcissism, *t*(304) = -0.52, *p* = .661, suggesting that individuals high in vulnerable narcissism do not, overall, view their status as meeting their aspirations. Inclusion aspirations became moderately negatively related to grandiose narcissism, *t*(304) = −4.99, *p* < .0001, and entitlement, *t*(303) = −3.17, *p* = .002, suggesting that their zero-order correlations with both reflect variance shared between inclusion aspirations and status aspirations. Individuals high in grandiose narcissism and entitlement may seek inclusion largely as a means to enhance status. Inclusion aspirations, however, remained moderately positively related to vulnerable narcissism, *t*(304) = 6.27, *p* < .0001. In contrast, perceived inclusion attainment related moderately negatively to all expressions of narcissism, all *t* < −3.20, all *p* < .001.

### Discussion

Grandiose narcissism, entitlement, and vulnerable narcissism all related positively to aspirations for status. In contrast, grandiose narcissism and entitlement, but not vulnerable narcissism, related positively to perceptions of higher status attainment. This pattern of results suggests that individuals high in grandiose narcissism generally perceive themselves to have high status, but those high in vulnerable narcissism do not, especially in relation to their aspirations. In contrast, individuals high in grandiose narcissism and entitlement did not aspire to higher inclusion once aspirations for status were controlled, suggesting that they may seek inclusion only insofar as they feel it helps them achieve status. They also did not feel they had attained inclusion, once perceptions of status were controlled. These results are consistent with previous research showing that those high in grandiose narcissism orient toward agency over communion (Campbell & Foster, 2007; Giacomin & Jordan, 2014, 2016; Zeigler-Hill et al., 2019). Those high in vulnerable narcissism also did not feel they had attained high inclusion, but they did aspire to high inclusion, even with aspirations for status controlled. Thus, vulnerable narcissism was associated with desires for both status and inclusion, but a perceived lack of attainment of both, particularly in relation to their high aspirations.

## Study 2

Study 2 replicated and extended Study 1 using alternative measures of grandiose and vulnerable narcissism. As in Study 1, it examined the associations between people’s aspirations for status and inclusion, their perceived attainment of status and inclusion, and their levels of grandiose and vulnerable narcissism. In addition, Study 2 more specifically examined how aspirations for, and perceived attainment of, status and inclusion relate to the three dimensions identified by the trifurcated model of narcissism (Miller et al., 2017), namely, agentic extraversion, antagonism, and narcissistic neuroticism.

Agentic extraversion is unique to grandiose narcissism and relates closely to the NSM dimension of grandiosity. Narcissistic neuroticism is unique to vulnerable narcissism and relates closely to the NSM dimension of vulnerability. The trifurcated model and NSM differ in their specification of the dimension common to grandiose and vulnerable narcissism, with the NSM specifying the narrow dimension of self-important entitlement, and the trifurcated model specifying the broader dimension of antagonism. In Study 2, we selected measures that provide a strong representation of both grandiose and vulnerable narcissism, as well as agentic extraversion, antagonism, and narcissistic neuroticism.

### Method

#### Procedure

Study 2 followed the same procedure as Study 1. It was hosted online on *Qualtrics*^TM^, and participants were recruited via *Amazon Mechanical Turk*^TM^ for a fee. After indicating informed consent, participants completed the study online.

#### Participants

A total of 441 attempts were logged for the study. Of these, we excluded 74 for the same reasons as Study 1. The final sample comprised 367 participants (220 men, 141 women, one unspecified, and four who did not respond) who ranged in age from 18 to 74 years (*M* = 35.90, *SD =* 10.80). Participants predominantly identified their backgrounds as White/European (58.9%), followed by Black/African/Caribbean (31.3%), Southeast Asian (2.5%), South Asian (2.5%), Latin American (2.5%) and Other (1.4%).

### Measures

Status aspirations, inclusion aspirations, perceived status attainment, and perceived inclusion attainment were measured as in Study 1.

#### Narcissism

The different expressions of narcissism were assessed using three measures: the *Narcissistic Admiration and Rivalry Questionnaire* (NARQ; Back et al., 2013), the short version of the *Five Factor Narcissism Inventory* (FFNI-SF; Sherman et al., 2015), and the *Hypersensitive Narcissism Scale* (HSNS; Hendin & Cheek, 1997).

The 18-item NARQ assesses grandiose narcissism along two broad dimensions: narcissistic admiration and narcissistic rivalry. The former reflects assertive self-enhancement (e.g., “I am great”), whereas the latter reflects antagonistic self-protection (e.g., “I want my rivals to fail”). Participants indicated the extent to which each statement described them (1 = *not agree at all*, 6 = *agree completely*). Narcissistic admiration reflects agentic extraversion, whereas narcissistic rivalry reflects antagonism (Crowe et al., 2019).

The 60-item FFNI-SF assesses both grandiose and vulnerable narcissism (e.g., “I only associate with people of my caliber”). It assesses 15 facets of narcissism. Scores on these facets can be combined to create subscales that assess grandiose narcissism (based on indifference, exhibitionism, authoritativeness, grandiose fantasies, manipulativeness, exploitativeness, entitlement, lack of empathy, arrogance, acclaim seeking, and thrill seeking) and vulnerable narcissism (based on reactive anger, shame, need for admiration, and distrust), or, alternatively, the three dimensions of the trifurcated model; specifically agentic extraversion (based on acclaim seeking, authoritativeness, grandiose fantasies, and exhibitionism), antagonism (based on manipulativeness, exploitativeness, entitlement, lack of empathy, arrogance, reactive anger, distrust, and thrill seeking), and narcissistic neuroticism (based on shame, indifference [reversed] and need for admiration). Participants indicated the extent to which each statement described them (1 = *disagree strongly*, 5 = *agree strongly*).

The 10-item HSNS assesses vulnerable narcissism (e.g., “I am secretly ‘put out’ or annoyed when other people come to me with their troubles, asking me for my time and sympathy”). Participants indicated the extent to which each statement described them (1 = *very uncharacteristic, strongly disagree*, 5 = *very characteristic or true, strongly agree*).^2^

### Results

Tables 3 and 4 show the descriptive statistics, internal consistencies, and zero-order correlations for all variables. All measures showed good internal consistency. As in Study 1, aspirations for status correlated positively with aspirations for inclusion, and perceived attainment of status correlated positively with perceived attainment of inclusion. Aspirations for status again correlated positively with perceived attainment of status, whereas aspirations for inclusion were uncorrelated with perceived attainment of inclusion.^3^

**Table 3.** Study 2: Descriptive Statistics and Inter-Correlations for the Main Variables.

Variable	1	2	3	4	5	6	7
1. Status aspirations	1	—	—	—	—	—	—
2. Inclusion aspirations	.73***	1	—	—	—	—	—
3. Perceived status attainment	.45***	.24**	1	—	—	—	—
4. Perceived inclusion attainment	.13*	.04	.69***	1	—	—	—
5. Grandiose narcissism (FFNI)	.78***	.69***	.48***	.11*	1	—	—
6. Vulnerable narcissism (FFNI)	.67***	.71***	−.03	−.26***	.63***	1	—
7. Vulnerable narcissism (HSNS)	.71***	.69***	−.08	−.20***	.69***	.85***	1
*Mean*	3.20	3.21	3.69	3.89	2.93	2.94	3.09
*SD*	.95	.96	.85	.76	.92	.86	.91
*Cronbach’s alpha*	.90	.92	.90	.89	.97	.91	.88

*Note*. We assessed status aspirations and inclusion aspirations using two structurally validated questionnaires developed by Mahadevan et al. (2019b). We assessed perceived status attainment and perceived inclusion attainment using two structurally validated questionnaires adapted from Huo et al. (2010). We assessed grandiose narcissism using the Narcissistic Admiration and Rivalry Questionnaire (Back et al., 2013) and the grandiosity subscale of the short version of the Five-Factor Narcissism Inventory (Sherman et al., 2015). We assessed vulnerable narcissism using the vulnerability subscale of the short version of the Five-Factor Narcissism Inventory (Sherman et al., 2015) and the Hypersensitive Narcissism Scale (Hendin & Cheek, 1997).

* *p* < .05, **p < .01, ***p < .001.

**Table 4.** Study 2: Descriptive Statistics and Inter-Correlations for the Trifurcated Model Dimensions.

Variable	1	2	3	4	5	6	7	8	9
1. Status aspirations	1	–	–	–	–	–	–	–	–
2. Inclusion aspirations	.73***	1	–	–	–	–		–	–
3. Perceived status attainment	.45***	.24**	1	–	–	–	–	–	–
4. Perceived inclusion attainment	.13*	.04	.69***	1	–	–	–	–	–
5. FFNI-Extraversion	.75***	.51***	.61***	.26***	1	–	–	–	–
6. NARQ-Admiration	.75***	.52***	.64***	.31***	.86***	1	–	–	–
7. FFNI-Antagonism	.76***	.65***	.30**	−.05	.72***	.71***	1	–	–
8. NARQ-Rivalry	.69***	.66***	.19**	−.13*	.57***	.64***	.89***	1	–
9. FFNI-Neuroticism	.53***	.71***	−.16**	−.29***	.22**	.25**	.54***	.64***	1
*Mean*	3.20	3.21	3.69	3.89	3.20	3.76	2.74	3.02	2.95
*SD*	.95	.96	.85	.76	.86	1.22	1.02	1.47	.86
*Cronbach’s alpha*	.90	.92	.90	.89	.91	.91	.97	.95	.80

*Note*. We assessed status aspirations and inclusion aspirations using two structurally validated questionnaires developed by Mahadevan et al. (2019b). We assessed perceived status attainment and perceived inclusion attainment using two structurally validated questionnaires adapted from Huo et al. (2010). We assessed grandiose narcissism using the Narcissistic Admiration and Rivalry Questionnaire (Back et al., 2013) and the grandiosity subscale of the short version of the Five Factor Narcissism Inventory (Sherman et al., 2015). We assessed vulnerable narcissism using the vulnerability subscale of the short version of the Five-Factor Narcissism Inventory (Sherman et al., 2015) and the Hypersensitive Narcissism Scale (Hendin & Cheek, 1997).

* *p* < .05, **p < .01, ***p < .001.

#### Zero-order correlations

We began by examining the zero-order correlations between aspirations for status and inclusion, perceived attainment of status and inclusion, and all expressions of narcissism (Tables 3 and 4). For each set of analyses, we first describe results for grandiose and vulnerable narcissism; then we describe results for agentic extraversion, antagonism, and narcissistic neuroticism. It is important to bear in mind that these results are not independent in the case of FFNI subscales, as subscales reflecting two- and three-factor structures share items.

Status aspirations correlated strongly positively with both grandiose narcissism and vulnerable narcissism (Table 3). Perceived status attainment correlated strongly positively with grandiose narcissism, but was unrelated to vulnerable narcissism. Inclusion aspirations correlated strongly positively with both grandiose and vulnerable narcissism. Perceived inclusion attainment was modestly positively correlated with grandiose narcissism and modestly negatively correlated with vulnerable narcissism.

Agentic extraversion, antagonism, and narcissistic neuroticism all correlated strongly positively with status aspirations (Table 4). Perceived status attainment, however, correlated strongly positively with agentic extraversion and moderately positively with antagonism but slightly negatively with narcissistic neuroticism. Inclusion aspirations correlated strongly positively with all three expressions of narcissism, whereas perceived inclusion attainment correlated moderately positively with agentic extraversion, modestly negatively with antagonism (albeit non-significantly for FFNI-antagonism), and moderately negatively with narcissistic neuroticism.

#### Regression analyses

We next examined the unique associations of aspirations for status and inclusion, and perceived attainment of status and inclusion, with expressions of narcissism. We regressed each measure of narcissism separately onto all four predictors simultaneously. Notably, status aspirations remained strongly positively related to both grandiose narcissism, FFNI-grandiose *t*(362) = 11.38, *p* < .0001, and vulnerable narcissism, FFNI-vulnerable *t*(362) = 9.60, *p* < .0001; HSNS *t*(362) = 10.61, *p* < .0001; Table 5. Perceived status attainment remained moderately positively related to grandiose narcissism, FFNI-grandiose *t*(362) = 6.62, *p* < .0001, but became modestly negatively related to vulnerable narcissism, FFNI-vulnerable *t*(362) = −4.11, *p* < .0001; HSNS *t*(362) = −1.95, *p* = .05. Thus, those high in vulnerable narcissism did not perceive themselves to be high in status relative to their aspirations. Inclusion aspirations remained modestly positively related to grandiose narcissism, FFNI-grandiose *t*(362) = 2.26, *p* = .025, and moderately positively related to vulnerable narcissism, FFNI-vulnerable *t*(362) = 9.14, *p* < .0001; HSNS *t*(362) = 6.93, *p* < .0001. As in Study 1, perceived inclusion attainment was moderately negatively related to both grandiose narcissism, FFNI-grandiose *t*(362) = −4.49, *p* < .0001, and vulnerable narcissism, FFNI-vulnerable *t*(362) = −4.64, *p* < .0001; HSNS *t*(362) = −4.73, *p* < .0001, once perceived status attainment was controlled.

**Table 5.** Study 2: Standardized Regression Coefficients for Regression of Aspirations for, and Perceived Attainment of, Status and Inclusion on Grandiose and Vulnerable Narcissism.

Variable	Grandiose narcissism (FFNI)	Vulnerable narcissism (FFNI)	Vulnerable narcissism (HSNS)
Status aspirations	.58***	.48***	.55***
Inclusion aspirations	.10*	.41***	.32***
Perceived status attainment	.33***	−.20***	−.10*
Perceived inclusion attainment	−.20***	−.20***	−.21***

*Note*. We assessed status aspirations and inclusion aspirations using two structurally validated questionnaires developed by Mahadevan et al. (2019b). We assessed perceived status attainment and perceived inclusion attainment using two structurally validated questionnaires adapted from Huo et al. (2010). We assessed grandiose narcissism using the Narcissistic Admiration and Rivalry Questionnaire (Back et al., 2013) and the grandiosity subscale of the short version of the Five-Factor Narcissism Inventory (Sherman et al., 2015). We assessed vulnerable narcissism using the vulnerability subscale of the short version of the Five-Factor Narcissism Inventory (Sherman et al., 2015) and the Hypersensitive Narcissism Scale (Hendin & Cheek, 1997).

* *p* < .05, **p < .01, ***p < .001.

Status aspirations remained strongly positively related to agentic extraversion, FFNI-extraversion *t*(362) = 11.23, *p* < .0001; NARQ-admiration *t*(363) = 11.33, *p* < .0001, and antagonism, FFNI-antagonism *t*(362) = 10.87, *p* < .0001; NARQ-rivalry *t*(363) = 7.86. *p* < .0001, and moderately positively related to narcissistic neuroticism, FFNI-neuroticism *t*(362) = 4.70, *p* < .0001; Table 6. Perceived status attainment remained moderately positively related to agentic extraversion, FFNI-extraversion *t*(362) = 8.45, *p* < .0001; NARQ-admiration *t*(363) = 8.65, *p* < .0001; became modestly positively related to antagonism, FFNI-antagonism *t*(362) = 3.38, *p* < .0001; NARQ-rivalry *t*(362) = 1.69, *p* = .09; and became moderately negatively related to narcissistic neuroticism, FFNI-neuroticism *t*(362) = −6.67, *p* < .0001. Thus, controlling for status aspirations did not affect the perceived status attainment of those high in agentic extraversion, but tempered the perceived status attainment of those high in antagonism or, especially, narcissistic neuroticism.

**Table 6.** Study 2: Standardized Regression Coefficients for Regression of Aspirations for, and Perceived Attainment of, Status and Inclusion on the Trifurcated Model Dimensions.

Variable	Agentic extraversion	Self-centered antagonism		Narcissistic neuroticism	
	FFNI-Extraversion	NARQ-Admiration	FFNI-Antagonism	NARQ-Rivalry	FFNI-Neuroticism
Status aspirations	.57***	.56***	.57***	.45***	.24***
Inclusion aspirations	.01	.01	.20***	.32***	.61***
Perceived status attainment	.42***	.42***	.17*	.09	−.34***
Perceived inclusion attainment	−.10*	–.05	−.26***	–.26***	−.11*

*Note*. We assessed status aspirations and inclusion aspirations using two structurally validated questionnaires developed by Mahadevan et al. (2019b). We assessed perceived status attainment and perceived inclusion attainment using two structurally validated questionnaires adapted from Huo et al. (2010). We assessed grandiose narcissism using the Narcissistic Admiration and Rivalry Questionnaire (Back et al., 2013) and the grandiosity subscale of the short version of the Five-Factor Narcissism Inventory (Sherman et al., 2015). We assessed vulnerable narcissism using the vulnerability subscale of the short version of the Five-Factor Narcissism Inventory (Sherman et al., 2015) and the Hypersensitive Narcissism Scale (Hendin & Cheek, 1997).

* *p* < .05, **p < .01, ***p < .001.

Inclusion aspirations became unrelated to agentic extraversion, FFNI-extraversion *t*(362) = .31, *p* = .761; NARQ-admiration *t*(363) = .14, *p* = .892; remained positively, though modestly, related to antagonism, FFNI-antagonism *t*(362) = 4.22, *p* < .0001; NARQ-rivalry *t*(363) = 6.26. *p* < .0001; and remained strongly positively related to narcissistic neuroticism, FFNI-neuroticism *t*(362) = 13.24, *p* < .0001. Thus, controlling for status aspirations eliminated the relation of agentic extraversion to inclusion aspirations, but not the relation of antagonism or, especially, narcissistic neuroticism to inclusion aspirations. Finally, perceived inclusion attainment became moderately negatively related to antagonism, FFNI-antagonism *t*(362) = −5.60; NARQ-rivalry *t*(363) = −5.26, and modestly negatively related to agentic extraversion and narcissistic neuroticism, FFNI-extraversion *t*(362) = −2.34; FFNI-neuroticism *t*(362) = −2.44; all *p* < .0001, with the exception of the NARQ-admiration, which was not significantly related to inclusion attainment, *t*(363) = −1.06, *p* = .290. Thus, all aspects of narcissism related to lower perceived inclusion attainment once perceived status and inclusion aspirations were controlled.

### Discussion

The results of Study 2 replicated and extended those of Study 1 using alternative measures of narcissism. The findings of Study 2 were largely consistent with those of Study 1 for grandiose and vulnerable narcissism. Individuals high in grandiose narcissism strongly aspired to higher status and felt they had attained it. In contrast, they did not strongly aspire to higher inclusion (once aspirations for status were controlled) and did not feel they had attained it. However, the pattern of results was slightly different than in Study 1. Whereas in Study 1, grandiose narcissism was not related to inclusion aspirations after controlling status aspirations, in Study 2 it remained significantly related to inclusion aspirations, but quite modestly (*β* = .10). In both studies, however, the relation of grandiose narcissism to aspirations for status was clearly stronger than its relation to aspirations for inclusion in the regression analyses. Those high in vulnerable narcissism, in contrast, aspired to high status, but did not feel they had attained it. They also aspired to high inclusion, but did not feel they had attained it. Thus, overall, vulnerable narcissism was associated with aspirations for both status and inclusion, but a lack of perceived attainment of both. The relation of vulnerable narcissism to aspirations for status and inclusion were comparable in magnitude.

With respect to the trifurcated model, all three dimensions underlying grandiose and vulnerable narcissism related positively to aspirations for status, even controlling for aspirations for inclusion. The three dimensions, however, differed in their relations to perceived attainment of status, and aspirations for, and perceived attainment of, inclusion. Like grandiose narcissism, agentic extraversion was associated with higher perceived attainment of status but was not associated with higher aspirations for inclusion (once aspirations for status were controlled), or higher perceived attainment of inclusion (once perceived attainment of status was controlled). Like vulnerable narcissism, narcissistic neuroticism was related to higher aspirations for inclusion but lower perceived attainment of both status and inclusion.

Finally, antagonism reflected something of a mix of these two patterns of relations. Antagonism related positively to perceived attainment of status, but considerably less strongly when aspirations for status were controlled, suggesting that those high in antagonism do not view their attainment of status as commensurate with their aspirations. Antagonism was associated modestly with higher aspirations for inclusion; notably its independent relation with aspirations for status was considerably stronger than its independent relation with aspirations for inclusion. Antagonism was, however, consistently related to lower perceived attainment of inclusion. The modest relation between antagonism and aspirations for inclusion suggests that this specific dimension of narcissism was responsible for the relation between grandiose narcissism and inclusion aspirations in this study. Notably, this aspiration was particularly pronounced when inclusion attainment was controlled, suggesting that individuals high in antagonism may desire inclusion that is greater than the notably low levels of inclusion they perceive themselves to have attained.

## General Discussion

The wide variety of personality features that have been used to describe narcissism, across different theoretical and disciplinary perspectives, has been a long-standing source of controversy (Cain et al., 2008; Miller et al., 2017; Wink, 1991). In what sense can individuals who are arrogant, bold, assertive, and dominant, as well as those who are fragile, submissive, and introverted be considered to be narcissistic? Recent research has significantly advanced our understanding of the structural features shared by grandiose and vulnerable narcissism (Krizan & Herlache, 2018; Miller et al., 2017). The current findings extend this work by suggesting that, functionally, a strong motivation for status may be shared by narcissistic personality features across the full narcissism spectrum. Although prior research has suggested that a strong desire for status underlies grandiose narcissism (Grapsas et al., 2020; Mahadevan et al., 2016; Zeigler-Hill et al., 2019), the present studies find direct evidence that it also characterizes vulnerable narcissism. These studies may thus contribute to a better understanding of the fundamental character of narcissism.

### Implications

Our findings suggest that a desire for status characterizes all expressions of narcissism; however, they differ in how they relate to desire for inclusion and perceived attainment of status and inclusion. Although individuals high in all aspects of narcissism may desire status and strive to view themselves as superior to others, they differ in their perceptions of how well they are meeting these goals. These results have implications for understanding differences between expressions of narcissism. It has been widely suggested that individuals high in grandiose narcissism manage trade-offs between status and inclusion by seeking status (Grapsas et al., 2020; Zeigler-Hill et al., 2019), consistent with our findings. When we tested unique associations, grandiose narcissism (and agentic extraversion) related strongly to desire for status but only modestly or not at all to desire for inclusion. But this pattern did not characterize individuals high in vulnerable narcissism (and narcissistic neuroticism) who also reported strong desire for inclusion. Notably, individuals high in vulnerable narcissism (and narcissistic neuroticism) perceived themselves to have low status and inclusion.

This pattern of results is consistent with a possible temporal dynamic linking grandiose and vulnerable narcissism. Pincus and Lukowitsky (2010; see also Wright & Edershile, 2018) outline an enduring clinical belief that individuals high in narcissistic grandiosity experience periodic bouts of vulnerability (e.g., Gore & Widiger, 2016). These bouts of vulnerability may reflect times when narcissistic individuals perceive themselves to have low status. It is clear that individuals high in grandiose narcissism desire high status and perceive themselves to have attained it successfully. If this perception falters, however, and their perceived status is threatened, they may experience narcissistic vulnerability. To compensate for a perceived loss of status, and otherwise try to satisfy a strong need for external validation (Zeigler-Hill et al., 2019), individuals high in vulnerable narcissism may seek social inclusion (“any port in a storm”). Although our data cannot test these dynamics, they are worth further consideration.

Our findings may provide preliminary support for a model that could integrate vulnerable narcissism into models of the dynamic pursuit of status within grandiose narcissism. Grapsas et al. (2020) and Zeigler-Hill et al. (2019) both suggest that narcissistic admiration is associated with the perception of high status attainment, through self-enhancement, but that rivalry reflects a vigilance for threats to status that may be associated with lower perceived attainment of status. Zeigler-Hill et al. (2019) found that narcissistic rivalry related negatively with daily perceptions of status attainment, which contrasts with our findings that it relates positively (though modestly) with perceptions of status attainment overall. Consistent with the theorizing of Grapsas et al. and Zeigler-Hill et al., narcissistic rivalry may attune people to acute threats to status (evident in more situational, daily reports), leading them to defensively assert their status (e.g., through derogation of others), mitigating the impact of threats to status. But if perceived status nevertheless drops further, then narcissistic vulnerability may increase, leading to avoidance of competition and increased efforts to be included (even if their interests in inclusion are superficial).

This latter possibility is also consistent with the imperative function of self-regard in hierometer theory (Mahadevan et al., 2016, 2020) wherein self-regard is theorized to regulate status-seeking behavior such that high self-regard promotes greater assertiveness and low self-regard greater acquiescence. Narcissistic vulnerability may lead individuals to withdraw from competition for status, unless success is highly likely. Those high in vulnerable narcissism view themselves as having low status and have low self-esteem—this may impel them to behave less assertively resulting in lower status. This possibility is also consistent with findings that vulnerable narcissism relates positively to hypercompetiveness but not competitiveness (Luchner et al., 2011). Individuals high in vulnerable narcissism may feel compulsively competitive toward others and cannot help but compare themselves to others, but avoid competitive situations because they feel unable to compete successfully.

Combining these perspectives, grandiose narcissism and agentic extraversion (or admiration) may relate to assertive behavior and self-enhancement that supports perceptions of status. This is consistent with our findings that grandiose narcissism and agentic extraversion relate to strong desire for status and perceptions of status attainment. If these perceptions are threatened, antagonism (or rivalry) may be related to vigilance for these threats and increase efforts to maintain status through competition and derogation of others (Grapsas et al., 2020). This is consistent with our findings that antagonism relates to strong desire for status but weaker (though positive) perceptions of status attainment. If rivalry fails to protect status, narcissistic vulnerability may result, spurring a withdrawal from competition and greater desire for inclusion to compensate for low status. This is consistent with our findings that vulnerable narcissism and narcissistic neuroticism relate more weakly to desire for status and more strongly to desire for inclusion (than grandiose narcissism and agentic extraversion), but relate negatively to perceived attainment of both. These considerations go beyond the data we have collected, but are consistent with our findings.

Our findings may also have implications for three-factor models of narcissism. Both the NSM and trifurcated model identify similar unique components of grandiose (e.g., immodesty, assertiveness) and vulnerable (e.g., neuroticism) narcissism, but differ in how they specify their shared components. The NSM focuses on the narrow dimension of entitlement (Krizan & Herlache, 2018), whereas the trifurcated model focuses on the broader dimension of antagonism (Miller et al., 2017). Recent factor analytic evidence is more consistent with a broad factor of antagonism being shared by grandiose and vulnerable narcissism (Crowe et al., 2019). Our findings may also support this possibility. In Study 1, entitlement revealed results that were nearly identical to those of grandiose narcissism. In contrast, in Study 2, antagonism revealed results that were distinct from both grandiose and vulnerable narcissism (and their phenotype-specific components) and consistent with prior theorizing about the role of antagonism in regulating status. In the context of status and inclusion, antagonism reveals a more unique motivational profile, relative to grandiose and vulnerable narcissism, than does entitlement. Future research should replicate these findings and further test which underlying dimension—entitlement or antagonism—better reflects the common structural components of grandiose and vulnerable narcissism.

### Limitations

Our considerations of possible within-person dynamics of narcissism (outlined above) highlight one limitation of the present research. The cross-sectional, correlational nature of the data meant that the temporal order between constructs could not be established. For example, we cannot determine whether being narcissistic engenders desire for status or whether desire for status fuels narcissism. Some links may be bi-directional in nature. For instance, perceptions of successful status attainment may fuel grandiose narcissism, which in turn facilitates successful attainment of status in future, or at least the self-perception of it. Experimental research would be valuable to determine the temporal order and causal relations between the constructs. Such research would also be invaluable in testing the potential implications of this research for within-person variations in aspirations and perceptions of status and inclusion and expressions of narcissism (Mahadevan et al., 2020).

Another limitation involves the use of self-report to assess all constructs. Self-reports can be vulnerable to social desirability concerns, shared method variance, and demand characteristics (Podsakoff et al., 2003). For example, some participants might lack insight into their own narcissistic tendencies or be unwilling to admit they desire status or inclusion (Carlson et al., 2013). We can also only make firm conclusions about perceptions of status and inclusion attainment, not how expressions of narcissism relate to actual attainment of status and inclusion. Accordingly, future research should implement additional methods beyond self-report, such as peer or family reports (Vazire & Mehl, 2008). It should be noted, however, that perceptions of status and inclusion attainment are fairly accurate, and likely to be powerful motivators whether they correspond with actual status and inclusion attainment or not (Anderson et al., 2001, 2006; Fournier, 2009).

A further limitation concerns the representativeness of our samples. While reasonably diverse in terms of age and gender, our samples consisted of U.S. residents; therefore, this research could not address the role of cross-cultural differences (Henrich et al., 2010). Future research should examine how aspirations and perceived attainment of status and inclusion relate to different expressions of narcissism in other populations, particularly non-Western cultures, to investigate the cross-cultural generalizability of these findings.

## Conclusions

These limitations notwithstanding, our findings suggest that a strong desire for status may be a functional commonality underlying all expressions of narcissism. They indicate that grandiose and vulnerable narcissism are united by a common desire for status. Notably, all underlying components of grandiose and vulnerable narcissism related to a strong desire for status, not only their common components of antagonism or entitlement. However, whereas individuals high in grandiose narcissism see themselves as having successfully attained status, those high in vulnerable narcissism do not. In addition, consistent with previous research showing that grandiose narcissism is characterized by a preference for agency over communion, those high in grandiose narcissism both desire and see themselves as having attained status, but neither desire and nor see themselves as having attained inclusion. In contrast, this pattern did not emerge for individuals high in vulnerable narcissism. These individuals desired both status and inclusion, and felt they had attained neither.

## Supplemental Material

sj-docx-1-psp-10.1177_01461672211021189 – Supplemental material for Desperately Seeking Status: How Desires for, and Perceived Attainment of, Status and Inclusion Relate to Grandiose and Vulnerable Narcissism Click here for additional data file. Supplemental material, sj-docx-1-psp-10.1177_01461672211021189 for Desperately Seeking Status: How Desires for, and Perceived Attainment of, Status and Inclusion Relate to Grandiose and Vulnerable Narcissism by Nikhila Mahadevan and Christian Jordan in Personality and Social Psychology Bulletin

## Notes

1. We additionally included, in Study 1, the 10-item Rosenberg (1965) Self-Esteem Scale. The total item count for all measures was 148 items.

2. We additionally included, in Study 2, the 10-item Rosenberg (1965) Self-Esteem Scale and the 16-item Communal Narcissism Inventory (Gebauer et al., 2012). The total item count for all measures was 151 items.

3. In past research, aspirations for status and inclusion correlate positively, as do perceived status attainment and perceived inclusion attainment (Mahadevan et al., 2019b). In addition, status aspirations and perceived status attainment correlate positively, whereas inclusion aspirations and perceived inclusion attainment correlate negatively (Mahadevan et al., 2019b). Similar findings emerged in the present studies except that inclusion aspirations and perceived inclusion attainment were uncorrelated rather than negatively correlated. One possible explanation for these findings is that the drive for status operates in a consolidatory manner, whereas the drive for inclusion does not. That is, attaining or feeling that one has attained, high status may fuel a desire for more status leading to a positive relation between the two, whereas attaining or feeling that one has attained, high inclusion might not. Such a possibility is also consistent with hierometer theory which posits that higher status promotes higher self-regard, which in turn motivates greater assertiveness (Mahadevan et al., 2016, 2020).

## References

[bibr1-01461672211021189] Abele A. E. Wojciszke B. (2018). Agency and communion in social psychology. Routledge. 10.1037/0022-3514.93.5.751 17983298

[bibr2-01461672211021189] Anderson C. Hildreth J. A. D. Howland L. (2015). Is the desire for status a fundamental human motive? A review of the empirical literature. Psychological Bulletin, 141, 574–601. 25774679 10.1037/a0038781

[bibr3-01461672211021189] Anderson C. John O. P. Keltner D. Kring A. M. (2001). Who attains social status? Effects of personality and physical attractiveness in social groups. Journal of Personality and Social Psychology, 81, 116–132. 11474718 10.1037//0022-3514.81.1.116

[bibr4-01461672211021189] Anderson C. Kraus M. W. Galinsky A. D. Keltner D. (2012). The local-ladder effect: Social status and subjective well-being. Psychological Science, 23, 764–771. 22653798 10.1177/0956797611434537

[bibr5-01461672211021189] Anderson C. Srivastava S. Beer J. S. Spataro S. E. Chatman J. A. (2006). Knowing your place: Self-perceptions of status in face-to-face groups. Journal of Personality and Social Psychology, 91, 1094–1110. 17144767 10.1037/0022-3514.91.6.1094

[bibr6-01461672211021189] Back M. D. Küfner A. C. Dufner M. Gerlach T. M. Rauthmann J. F. Denissen J. J. (2013). Narcissistic admiration and rivalry: Disentangling the bright and dark sides of narcissism. Journal of Personality and Social Psychology, 105, 1013–1037. 24128186 10.1037/a0034431

[bibr7-01461672211021189] Baumeister R. F. Leary M. R. (1995). The need to belong: Desire for interpersonal attachments as a fundamental human motivation. Psychological Bulletin, 117, 497–529. 7777651

[bibr8-01461672211021189] Brown R. P. Zeigler-Hill V. (2004). Narcissism and the non-equivalence of self-esteem measures: A matter of dominance? Journal of Research in Personality, 38, 585–592.

[bibr9-01461672211021189] Cain N. M. Pincus A. L. Ansell E. B. (2008). Narcissism at the crossroads: Phenotypic description of pathological narcissism across clinical theory, social/personality psychology, and psychiatric diagnosis. Clinical Psychology Review, 28, 638–656. 18029072 10.1016/j.cpr.2007.09.006

[bibr10-01461672211021189] Campbell W. K. (1999). Narcissism and romantic attraction. Journal of Personality and Social Psychology, 77, 1254–1270.

[bibr11-01461672211021189] Campbell W. K. Bonacci A. M. Shelton J. Exline J. J. Bushman B. J. (2004). Psychological entitlement: Interpersonal consequences and validation of a self-report measure. Journal of Personality Assessment, 83, 29–45. 15271594 10.1207/s15327752jpa8301_04

[bibr12-01461672211021189] Campbell W. K. Foster J. D. (2007). The narcissistic self: Background, an extended agency model, and ongoing controversies. In Sedikides C. Spencer S. (Eds.), Frontiers in social psychology: The self (pp. 115–138). Psychology.

[bibr13-01461672211021189] Campbell W. K. Rudich E. A. Sedikides C. (2002). Narcissism, self-esteem, and the positivity of self-views: Two portraits of self-love. Personality and Social Psychology Bulletin, 28, 358–368.

[bibr14-01461672211021189] Carlson E. N. Vazire S. Oltmanns T. F. (2013). Self-other knowledge asymmetries in personality pathology. Journal of Personality, 81, 155–170. 22583054 10.1111/j.1467-6494.2012.00794.x PMC3424287

[bibr15-01461672211021189] Crowe M. L. Lynam D. R. Campbell W. K. Miller J. D. (2019). Exploring the structure of narcissism: Toward an integrated solution. Journal of Personality, 87, 1151–1169. 30742713 10.1111/jopy.12464

[bibr16-01461672211021189] Dickinson K. A. Pincus A. L. (2003). Interpersonal analysis of grandiose and vulnerable narcissism. Journal of Personality Disorders, 17, 188–207. 12839099 10.1521/pedi.17.3.188.22146

[bibr17-01461672211021189] Fiske S. T. (2010). Interpersonal stratification: Status, power, and subordination. In Fiske S. T. Gilbert D. T. Lindzey G. (Eds.), Handbook of social psychology (5th ed., pp. 941–982). Wiley.

[bibr18-01461672211021189] Foster J. D. Shrira I. Campbell W. K. (2006). Theoretical models of narcissism, sexuality, and relationship commitment. Journal of Social and Personal Relationships, 23, 367–386.

[bibr19-01461672211021189] Fournier M. A. (2009). Adolescent hierarchy formation and the social competition theory of depression. Journal of Social and Clinical Psychology, 28, 1144–1172.

[bibr20-01461672211021189] Gebauer J. E. Sedikides C. Verplanken B. Maio G. R. (2012). Communal narcissism. Journal of Personality and Social Psychology, 103, 854–878. 22889074 10.1037/a0029629

[bibr21-01461672211021189] Giacomin M. Jordan C. H. (2014). Down-regulating narcissistic tendencies: Communal focus reduces state narcissism. Personality and Social Psychology Bulletin, 40, 488–500. 24345714 10.1177/0146167213516635

[bibr22-01461672211021189] Giacomin M. Jordan C. H. (2016). The wax and wane of narcissism: Grandiose narcissism as a process or state. Journal of Personality, 84, 154–164. 25388437 10.1111/jopy.12148

[bibr23-01461672211021189] Gore W. L. Widiger T. A. (2016). Fluctuation between grandiose and vulnerable narcissism. Personality Disorders: Theory, Research, and Treatment, 7, 363–371. 10.1037/per0000181 26986960

[bibr24-01461672211021189] Grapsas S. Brummelman E. Back M. D. Denissen J. J. A. (2020). The “why” and “how” of narcissism: A process model of narcissistic status pursuit. Perspectives on Psychological Science, 15, 150–172. 31805811 10.1177/1745691619873350 PMC6970445

[bibr25-01461672211021189] Gregg A. P. Mahadevan N. (2014). Intellectual arrogance and intellectual humility: An evolutionary-epistemological account. Journal of Psychology and Theology, 42, 7–18.

[bibr26-01461672211021189] Gregg A. P. Mahadevan N. Sedikides C. (2017a). Intellectual arrogance and intellectual humility: Correlational evidence for an evolutionary-embodied-epistemological account. The Journal of Positive Psychology, 12, 59–73.

[bibr27-01461672211021189] Gregg A. P. Mahadevan N. Sedikides C. (2017b). The SPOT effect: People spontaneously prefer their own theories. Quarterly Journal of Experimental Psychology, 70, 996–1010. 10.1080/17470218.2015.1099162 26836058

[bibr28-01461672211021189] Gregg A. P. Mahadevan N. Sedikides C. (2018). Taking the high ground: The impact of social status on the derogation of ideological opponents. Social Cognition, 36, 43–77.

[bibr29-01461672211021189] Hendin H. M. Cheek J. M. (1997). Assessing hypersensitive narcissism: A reexamination of Murray’s Narcism Scale. Journal of Research in Personality, 31, 588–599.

[bibr30-01461672211021189] Henrich J. Heine S. J. Norenzayan A. (2010). The weirdest people in the world? Behavioral and Brain Sciences, 33, 61–83. 10.1017/S0140525X0999152X 20550733

[bibr31-01461672211021189] Hogan J. Holland B. (2003). Using theory to evaluate personality and job performance relations: A socioanalytic perspective. Journal of Applied Psychology, 88, 100–112. 10.1037/0021-9010.88.1.100 12675398

[bibr32-01461672211021189] Huo Y. J. Binning K. R. Molina L. E. (2010). Testing an integrative model of respect: Implications for social engagement and well-being. Personality and Social Psychology Bulletin, 36, 200–212. 20032268 10.1177/0146167209356787

[bibr33-01461672211021189] Krizan Z. Herlache A. D. (2018). The narcissism spectrum model: A synthetic view of narcissistic personality. Personality and Social Psychology Review, 22, 3–31. 28132598 10.1177/1088868316685018

[bibr34-01461672211021189] Luchner A. F. Houston J. M. Walker C. Houston M. A. (2011). Exploring the relationship between two forms of narcissism and competitiveness. Personality and Individual Differences, 51, 779–782.

[bibr35-01461672211021189] Mahadevan N. Gregg A. P. Sedikides C. (2019a). Is self-regard a sociometer or a hierometer? Self-esteem tracks status and inclusion, narcissism tracks status. Journal of Personality and Social Psychology, 116, 444–466. 29608073 10.1037/pspp0000189

[bibr36-01461672211021189] Mahadevan N. Gregg A. P. Sedikides C. (2019b). Where I am and where I want to be: Perceptions of and aspirations for status and inclusion differentially predict psychological health. Personality and Individual Differences, 139, 170–174.

[bibr37-01461672211021189] Mahadevan N. Gregg A. P. Sedikides C. (2020). The ups and downs of social life: Within-person variations in daily status and inclusion differentially predict self-regard and interpersonal behavior. Journal of Personality, 88, 1111–1128. 32432337 10.1111/jopy.12559

[bibr38-01461672211021189] Mahadevan N. Gregg A. P. Sedikides C. De Waal-Andrews W. (2016). Winners, losers, insiders, and outsiders: Comparing hierometer and sociometer theories of self-regard. Frontiers in Psychology, 7, 334. 27065896 10.3389/fpsyg.2016.00334 PMC4811877

[bibr39-01461672211021189] Miller J. D. Hoffman B. J. Gaughan E. T. Gentile B. Maples J. Keith Campbell W. (2011). Grandiose and vulnerable narcissism: A nomological network analysis. Journal of Personality, 79, 1013–1042. 21204843 10.1111/j.1467-6494.2010.00711.x

[bibr40-01461672211021189] Miller J. D. Lynam D. R. Hyatt C. S. Campbell W. K. (2017). Controversies in narcissism. Annual Review of Clinical Psychology, 13, 291–315. 10.1146/annurev-clinpsy-032816-045244 28301765

[bibr41-01461672211021189] Miller J. D. Price J. Gentile B. Lynam D. R. Campbell W. K. (2012). Grandiose and vulnerable narcissism from the perspective of the interpersonal circumplex. Personality and Individual Differences, 53, 507–512.

[bibr42-01461672211021189] Morf C. C. Rhodewalt F. (2001). Unraveling the paradoxes of narcissism: A dynamic self-regulatory processing model. Psychological Inquiry, 12, 177–196.

[bibr43-01461672211021189] Neel R. Kenrick D. T. White A. E. Neuberg S. L. (2016). Individual differences in fundamental social motives. Journal of Personality and Social Psychology, 110, 887–907. 26371400 10.1037/pspp0000068

[bibr44-01461672211021189] Nevicka B. (2018). Narcissism and leadership: A perfect match? In Hermann A. Brunell A. Foster J. (Eds.), Handbook of trait narcissism: Key advances, research methods, and controversies (pp. 399–408). New Press.

[bibr45-01461672211021189] Pincus A. L. Ansell E. B. Pimentel C. A. Cain N. M. Wright A. G. Levy K. N. (2009). Initial construction and validation of the pathological narcissism inventory. Psychological Assessment, 21, 365–379. 19719348 10.1037/a0016530

[bibr46-01461672211021189] Pincus A. L. Lukowitsky M. R. (2010). Pathological narcissism and narcissistic personality disorder. Annual Review of Clinical Psychology, 6, 421–446. 10.1146/annurev.clinpsy.121208.131215 20001728

[bibr47-01461672211021189] Podsakoff P. M. MacKenzie S. B. Lee J. Y. Podsakoff N. P. (2003). Common method biases in behavioral research: A critical review of the literature and recommended remedies. Journal of Applied Psychology, 88, 879–903. 10.1037/0021-9010.88.5.879 14516251

[bibr48-01461672211021189] Raskin R. Terry H. (1988). A principal-components analysis of the Narcissistic Personality Inventory and further evidence of its construct validity. Journal of Personality and Social Psychology, 54, 890–902. 3379585 10.1037//0022-3514.54.5.890

[bibr49-01461672211021189] Rogoza R. Cieciuch J. Strus W. Baran T. (2019). Seeking a common framework for research on narcissism: An attempt to integrate the different faces of narcissism within the Circumplex of Personality Metatraits. European Journal of Personality, 33, 437–455.

[bibr50-01461672211021189] Rosenberg M. (1965). Society and the adolescent self-image. Princeton University Press.

[bibr51-01461672211021189] Santor D. A. Messervey D. Kusumakar V. (2000). Measuring peer pressure, popularity, and conformity in adolescent boys and girls: Predicting school performance, sexual attitudes, and substance abuse. Journal of Youth and Adolescence, 29, 163–182.

[bibr52-01461672211021189] Schönbrodt F. D. Perugini M. (2013). At what sample size do correlations stabilize? Journal of Research in Personality, 47, 609–612.

[bibr53-01461672211021189] Sheldon K. M. Schüler J. (2011). Wanting, having, and needing: Integrating motive disposition theory and self-determination theory. Journal of Personality and Social Psychology, 101, 1106–1123. 21875226 10.1037/a0024952

[bibr54-01461672211021189] Sherman E. D. Miller J. D. Few L. R. Campbell W. K. Widiger T. A. Crego C. Lynam D. R. (2015). Development of a short form of the five-factor narcissism inventory: The FFNI-SF. Psychological Assessment, 27, 1110–1116. 25774640 10.1037/pas0000100

[bibr55-01461672211021189] Stillman T. F. Baumeister R. F. Lambert N. M. Crescioni A. W. DeWall C. N. Fincham F. D. (2009). Alone and without purpose: Life loses meaning following social exclusion. Journal of Experimental Social Psychology, 45, 686–694. 20161218 10.1016/j.jesp.2009.03.007 PMC2717555

[bibr56-01461672211021189] Twenge J. M. Baumeister R. F. Tice D. M. Stucke T. S. (2001). If you can’t join them, beat them: Effects of social exclusion on aggressive behavior. Journal of Personality and Social Psychology, 81, 1058–1069. 11761307 10.1037//0022-3514.81.6.1058

[bibr57-01461672211021189] Twenge J. M. Campbell W. K. (2003). “Isn’t it fun to get the respect that we’re going to deserve?” Narcissism, social rejection, and aggression. Personality and Social Psychology Bulletin, 29, 261–272. 15272953 10.1177/0146167202239051

[bibr58-01461672211021189] Vazire S. Mehl M. R. (2008). Knowing me, knowing you: The accuracy and unique predictive validity of self-ratings and other-ratings of daily behavior. Journal of Personality and Social Psychology, 95, 1202–1216. 18954202 10.1037/a0013314

[bibr59-01461672211021189] Weiss B. Campbell W. K. Lynam R. D. Miller J. D. (2019). A trifurcated model of narcissism: On the pivotal role of trait Antagonism. In Miller J. D. Lynam D. R. (Eds.), The handbook of antagonism: Conceptualizations, assessment, consequences, and treatment of the low end of Agreeableness (pp. 221–235). Academic Press.

[bibr60-01461672211021189] Wink P. (1991). Two faces of narcissism. Journal of Personality and Social Psychology, 61, 590–597. Academic Press. 1960651 10.1037//0022-3514.61.4.590

[bibr61-01461672211021189] Wright A. G. Edershile E. A. (2018). Issues resolved and unresolved in pathological narcissism. Current Opinion in Psychology, 21, 74–79. 29059578 10.1016/j.copsyc.2017.10.001

[bibr62-01461672211021189] Zeigler-Hill V. Vrabel J. K. McCabe G. A. Cosby C. A. Traeder C. K. Hobbs K. A. Southard A. C. (2019). Narcissism and the pursuit of status. Journal of Personality, 87, 310–327. 29637567 10.1111/jopy.12392

