# Supplementary material for: Desperately Seeking Status: How Desires for, and Perceived Attainment of, Status and Inclusion Relate to Grandiose and Vulnerable Narcissism
Source: Pers Soc Psychol Bull. 2021 Jun 11;48(5):704–17. doi: 10.1177/01461672211021189 (PMC9066682; doi:10.1177/01461672211021189)
Supplement: sj-docx-1-psp-10.1177_01461672211021189 – Supplemental material for Desperately Seeking Status: How Desires for, and Perceived Attainment of, Status and Inclusion Relate to Grandiose and Vulnerable Narcissism [file sj-docx-1-psp-10.1177_01461672211021189.docx]

Study 1

Please select the most appropriate response for each of the following items.

Most of the time I feel that people...

|  | 1 (Disagree strongly) | 2 (Disagree a little) | 3 (Neither agree nor disagree) | 4 (Agree a little) | 5 (Agree strongly) |
| --- | --- | --- | --- | --- | --- |
| ... like me as a person |  |  |  |  |  |
| ... feel warmly toward me |  |  |  |  |  |
| ... consider me to be a nice person to have around |  |  |  |  |  |
| ... don't like me |  |  |  |  |  |
| ... include me in their social activities |  |  |  |  |  |
| ... are happy for me to belong to their social groups |  |  |  |  |  |
| ... accept me |  |  |  |  |  |
| ... see me as fitting in |  |  |  |  |  |
| ... would be willing to be friends with me |  |  |  |  |  |

Please select the most appropriate response for each of the following items.

Most of the time I feel that people...

|  | 1 (Disagree strongly) | 2 (Disagree a little) | 3 (Neither agree nor disagree) | 4 (Agree a little) | 5 (Agree strongly) |
| --- | --- | --- | --- | --- | --- |
| ... respect my achievements |  |  |  |  |  |
| ...value my opinions and ideas |  |  |  |  |  |
| ...think highly of my abilities and talents |  |  |  |  |  |
| ...admire me |  |  |  |  |  |
| ...consider me a success |  |  |  |  |  |
| ...look up to me |  |  |  |  |  |
| ...see me as an important person |  |  |  |  |  |
| ...consider me a high-status individual |  |  |  |  |  |

Please select the most appropriate response for each of the following items.

|  | 1 (Strongly disagree) | 2 (Disagree a little) | 3 (Neither agree nor disagree) | 4 (Agree a little) | 5 (Agree strongly) |
| --- | --- | --- | --- | --- | --- |
| Above all, I want to be accepted. |  |  |  |  |  |
| Being liked by others is essential to me. |  |  |  |  |  |
| Belonging to social groups, and being included in their activities, means everything to me. |  |  |  |  |  |
| I desire, first and foremost, to have many friends and close relationships. |  |  |  |  |  |
| Fitting in with others really matters to me. |  |  |  |  |  |
| Even mild rejection really bothers me. |  |  |  |  |  |
| I hate it when other people don't warm up to me. |  |  |  |  |  |
| I fear not fitting in, or being someone others are indifferent to. |  |  |  |  |  |
| Being disliked by others is the worst thing in the world. |  |  |  |  |  |
| I dread nothing more than people excluding me from their social circle. |  |  |  |  |  |

Please select the appropriate response for each of the following items.

|  | 1 (Disagree strongly) | 2 (Disagree a little) | 3 (Neither agree nor disagree) | 4 (Agree a little) | 5 (Agree strongly) |
| --- | --- | --- | --- | --- | --- |
| Above all, I want to be successful. |  |  |  |  |  |
| Having the respect of others is essential to me. |  |  |  |  |  |
| Being admired for my talents, and recognized for achievements, means everything to me. |  |  |  |  |  |
| I aspire, first and foremost, to be a person of importance and distinction. |  |  |  |  |  |
| Getting ahead of the competition really matters to me. |  |  |  |  |  |
| Just being average really bothers me. |  |  |  |  |  |
| I hate it when other people don't acknowledge my potential. |  |  |  |  |  |
| I fear being insignificant, or being just another face in the crowd. |  |  |  |  |  |
| Being disrespected by others is the worst thing in the world. |  |  |  |  |  |
| I dread nothing more than being left behind in the struggle to survive. |  |  |  |  |  |

This inventory consists of a number of pairs of statements with which you may or may not identify.   Consider this example: A. I like having authority over people B. I don't mind following orders   Which of these two statements is closer to your own feelings about yourself?  If you identify more with "liking to have authority over people" than with "not minding following orders", then you would choose option A.   You may identify with both A and B.  In this case you should choose the statement which seems closer to yourself.  Or, if you do not identify with either statement, select the one which is least objectionable or remote.  In other words, read each pair of statements and then choose the one that is closer to your own feelings.  Indicate your answer by writing the letter (A or B) in the space provided to the right of each item.  Please do not skip any items.

NPI1 Select one of the following:

- A. I have a natural talent for influencing people.
- B. I am not good at influencing people.

NPI2 Select one of the following:

- A. Modesty doesn't become me.
- B. I am essentially a modest person.

NPI3 Select one of the following:

- A. I would do almost anything on a dare.
- B. I tend to be a fairly cautious person.

NPI4 Select one of the following:

- A. When people compliment me I sometimes get embarrassed.
- B. I know that I am good because everybody keeps telling me so.

NPI5 Select one of the following:

- A. The thought of ruling the world frightens the hell out of me.
- B. If I ruled the world it would be a better place.

NPI6 Select one of the following:

- A. I can usually talk my way out of anything.
- B. I try to accept the consequences of my behavior.

NPI7 Select one of the following:

- A. I prefer to blend in with the crowd.
- B. I like to be the center of attention.

NPI8 Select one of the following:

- A. I will be a success.
- B. I am not too concerned about success.

NPI9 Select one of the following:

- A. I am no better or worse than most people.
- B. I think I am a special person.

NPI10 Select one of the following:

- A. I am not sure if I would make a good leader.
- B. I see myself as a good leader.

NPI11 Select one of the following:

- A. I am assertive.
- B. I wish I were more assertive.

NPI12 Select one of the following:

- A. I like to have authority over other people.
- B. I don't mind following orders.

NPI13 Select one of the following:

- A. I find it easy to manipulate people.
- B. I don't like it when I find myself manipulating people.

NPI14 Select one of the following:

- A. I insist upon getting the respect that is due me.
- B. I usually get the respect that I deserve.

NPI15 Select one of the following:

- A. I don't particularly like to show off my body.
- B. I like to show off my body.

NPI16 Select one of the following:

- A. I can read people like a book.
- B. People are sometimes hard to understand.

NPI17 Select one of the following:

- A. If I feel competent I am willing to take responsibility for making decisions.
- B. I like to take responsibility for making decisions.

NPI18 Select one of the following:

- A. I just want to be reasonably happy.
- B. I want to amount to something in the eyes of the world.

NPI19 Select one of the following:

- A. My body is nothing special.
- B. I like to look at my body.

NPI20 Select one of the following:

- A. I try not to be a show off.
- B. I will usually show off if I get the chance.

NPI21 Select one of the following:

- A. I always know what I am doing.
- B. Sometimes I am not sure of what I am doing.

NPI22 Select one of the following:

- A. I sometimes depend on people to get things done.
- B. I rarely depend on anyone else to get things done.

NPI23 Select one of the following:

- A. Sometimes I tell good stories.
- B. Everybody likes to hear my stories.

NPI24 Select one of the following:

- A. I expect a great deal from other people.
- B. I like to do things for other people.

NPI25 Select one of the following:

- A. I will never be satisfied until I get all that I deserve.
- B. I take my satisfactions as they come.

NPI26 Select one of the following:

- A. Compliments embarrass me.
- B. I like to be complimented.

NPI27 Select one of the following:

- A. I have a strong will to power.
- B. Power for its own sake doesn't interest me.

NIP28 Select one of the following:

- A. I don't care about new fads and fashions.
- B. I like to start new fads and fashions.

NPI29 Select one of the following:

- A. I like to look at myself in the mirror.
- B. I am not particularly interested in looking at myself in the mirror.

NPI30 Select one of the following:

- A. I really like to be the center of attention.
- B. It makes me uncomfortable to be the center of attention.

NPI31 Select one of the following:

- A. I can live my life in any way I want to.
- B. People can't always live their lives in terms of what they want.

NPI32 Select one of the following:

- A. Being an authority doesn't mean that much to me.
- B. People always seem to recognize my authority.

NPI33 Select one of the following:

- A. I would prefer to be a leader.
- B. It makes little difference to me whether I am a leader or not.

NPI34 Select one of the following:

- A. I am going to be a great person.
- B. I hope I am going to be successful.

NPI35 Select one of the following:

- A. People sometimes believe what I tell them.
- B. I can make anybody believe anything I want them to.

NPI36 Select one of the following:

- A. I am a born leader.
- B. Leadership is a quality that takes a long time to develop.

NPI37 Select one of the following:

- A. I wish somebody would someday write my biography.
- B. I don't like people to pry into my life for any reason.

NPI38 Select one of the following:

- A. I get upset when people don't notice how I look when I go out in public.
- B. I don't mind blending into the crowd when I go out in public.

NPI39 Select one of the following:

- A. I am more capable than other people.
- B. There is a lot that I can learn from other people.

NPI40 Select one of the following:

- A. I am much like everybody else.
- B. I am an extraordinary person.

Below you will find 52 descriptive statements. Please consider each one and indicate how well that statement describes you. Please respond to all statements. There are no right or wrong answers. Simply indicate how well each statement describes you as a person using the following scale:

|  | Not at all like me | Moderately unlike me | A little unlike me | A little like me | Moderately like me | Very much like me |
| --- | --- | --- | --- | --- | --- | --- |
| I often fantasize about being admired and respected. |  |  |  |  |  |  |
| My self-esteem fluctuates a lot. |  |  |  |  |  |  |
| I sometimes feel ashamed about my expectations of others when they disappoint me. |  |  |  |  |  |  |
| I can usually talk my way out of anything. |  |  |  |  |  |  |
| It's hard to feel good about myself when I'm alone. |  |  |  |  |  |  |
| I can make myself feel good by caring for others. |  |  |  |  |  |  |
| I hate asking for help. |  |  |  |  |  |  |
| When people don't notice me, I start to feel bad about myself. |  |  |  |  |  |  |
| I often hide my needs for fear that others will see me as needy and dependent. |  |  |  |  |  |  |
| I can make anyone believe anything I want them to. |  |  |  |  |  |  |
| I get mad when people don't notice all that I do for them. |  |  |  |  |  |  |
| I get annoyed by people who are not interested in what I say or do. |  |  |  |  |  |  |
| I wouldn't disclose all my intimate thoughts and feelings to someone I didn't admire. |  |  |  |  |  |  |
| I often fantasize about having a huge impact on the world around me. |  |  |  |  |  |  |
| I find it easy to manipulate people |  |  |  |  |  |  |
| When other don't notice me, I start to feel worthless. |  |  |  |  |  |  |
| Sometimes I avoid people because I'm concerned they'll disappoint me. |  |  |  |  |  |  |
| I typically get very angry when I'm unable to get what I want from others. |  |  |  |  |  |  |
| I sometimes need important others in my life to reassure me of my self-worth. |  |  |  |  |  |  |
| When I do things for other people, I expect them to do things for me. |  |  |  |  |  |  |
| When others don't meet my expectations, I often feel ashamed about what I wanted. |  |  |  |  |  |  |
| I feel important when others rely on me. |  |  |  |  |  |  |
| I can read people like a book. |  |  |  |  |  |  |
| When others disappoint me, I often get angry at myself. |  |  |  |  |  |  |
| Sacrificing for others makes me the better person. |  |  |  |  |  |  |
| I often fantasize about accomplishing things that are probably beyond my means. |  |  |  |  |  |  |
| Sometimes I avoid people because I'm afraid they won't do what I want them to. |  |  |  |  |  |  |
| It's hard to show others the weaknesses I feel inside. |  |  |  |  |  |  |
| I get angry when criticized. |  |  |  |  |  |  |
| It's hard to feel good about myself unless I know other people admire me. |  |  |  |  |  |  |
| I often fantasize about being rewarded for my efforts. |  |  |  |  |  |  |
| I am preoccupied with thoughts and concerns that most people are not interested in me. |  |  |  |  |  |  |
| I like to have friends who rely on me because it makes me feel important. |  |  |  |  |  |  |
| Sometimes I avoid people because I'm concerned they won't acknowledge what I do for them. |  |  |  |  |  |  |
| Everybody likes to hear my stories. |  |  |  |  |  |  |
| It's hard for me to feel good about myself unless I know other people like me. |  |  |  |  |  |  |
| It irritates me when people don't notice how good a person I am. |  |  |  |  |  |  |
| I will never be satisfied until I get all that I deserve. |  |  |  |  |  |  |
| I try to show what a good person I am through my sacrifices. |  |  |  |  |  |  |
| I am disappointed when people don't notice me. |  |  |  |  |  |  |
| I often find myself envying others' accomplishments. |  |  |  |  |  |  |
| I often fantasize about performing heroic deeds. |  |  |  |  |  |  |
| I help others in order to prove I'm a good person. |  |  |  |  |  |  |
| It's important to show people I can do it on my own, even if I have some doubts inside. |  |  |  |  |  |  |
| I often fantasize about being recognized for my accomplishments. |  |  |  |  |  |  |
| I can't stand relying on other people because it makes me feel weak. |  |  |  |  |  |  |
| When others don't respond to me the way that I would like them to, it is hard for me to still feel ok with myself. |  |  |  |  |  |  |
| I need others to acknowledge me. |  |  |  |  |  |  |
| I want to amount to something in the eyes of the world. |  |  |  |  |  |  |
| When others get a glimpse of my needs, I feel anxious and ashamed. |  |  |  |  |  |  |
| Sometimes it's easier to be alone than to face not getting everything I want from other people. |  |  |  |  |  |  |
| I can get pretty angry when others disagree with me. |  |  |  |  |  |  |

Please respond to the following items by selecting the number that best reflects your own beliefs.

|  | 1 (Strongly Disagree) | 2 | 3 | 4 (Neither Agree nor Disagree) | 5 | 6 | 7 (Strongly Agree) |
| --- | --- | --- | --- | --- | --- | --- | --- |
| I honestly feel I'm just more deserving than others. |  |  |  |  |  |  |  |
| Great things should come to me. |  |  |  |  |  |  |  |
| If I were on the Titanic, I would deserve to be on the first life boat! |  |  |  |  |  |  |  |
| I demand the best because I'm worth it. |  |  |  |  |  |  |  |
| I do not necessarily deserve special treatment. |  |  |  |  |  |  |  |
| I deserve more things in my life. |  |  |  |  |  |  |  |
| People like me deserve an extra break now and then. |  |  |  |  |  |  |  |
| Things should go my way. |  |  |  |  |  |  |  |
| I feel entitled to more of everything. |  |  |  |  |  |  |  |

Below is a list of statements dealing with your general feelings about yourself. Please respond using the following scale.

|  | Strongly Disagree 1 | 2 | 3 | Neither Agree nor Disagree 4 | 5 | 6 | Strongly Agree 7 |
| --- | --- | --- | --- | --- | --- | --- | --- |
| On the whole, I am satisfied with myself. |  |  |  |  |  |  |  |
| At times, I think I am no good at all. |  |  |  |  |  |  |  |
| I feel that I have a number of good qualities. |  |  |  |  |  |  |  |
| I am able to do things as well as most other people. |  |  |  |  |  |  |  |
| I feel I do not have much to be proud of. |  |  |  |  |  |  |  |
| I certainly feel useless at times. |  |  |  |  |  |  |  |
| I feel that I’m a person of worth, at least on an equal plane with others. |  |  |  |  |  |  |  |
| I wish I could have more respect for myself. |  |  |  |  |  |  |  |
| All in all, I am inclined to feel that I am a failure. |  |  |  |  |  |  |  |
| I take a positive attitude toward myself. |  |  |  |  |  |  |  |

Please fill in the demographic questions below. This information is helpful to ensure that we have a representative sample of participants in our study.

Age:

________________________________________________________________

Gender (select one):

- Male
- Female
- Other
- Rather not specify

Which of the following BEST describes your background?

- Aboriginal/First Nations/Metis
- White/European
- Black/African/Caribbean
- Southeast Asian (e.g., Chinese, Japanese, Korean, Vietnamese, Cambodian, Filipino, etc.)
- Arab (Saudi Arabian, Palestinian, Iraqi, etc.)
- South Asian (East Indian, Sri Lankan, etc.)
- Latin American (Costa Rican, Guatemalan, Brazilian, Columbian, etc.)
- West Asian (Iranian, Afghani, etc.)
- Other

How good is your English?

- Excellent
- Good
- Average
- Poor
- Very Poor

What do you think this study was about?

________________________________________________________________

When conducting research, we rely on participants' responses being honest and accurate in order for us to draw valid conclusions from the data. However, we recognize that there are many reasons participants might be unable or unwilling to provide fully honest and accurate responses. In these cases it is truly helpful for us to be able to identify responses that may not be valid so we can take this into account.
 
In your honest opinion, should we use your data from this survey? 
  
(Please note your answer is **confidential and you will be compensated** whichever answer you choose.)

- Yes
- No

If you selected "No", please specify: Why do you think we should **NOT** use your data?

________________________________________________________________

________________________________________________________________

________________________________________________________________

________________________________________________________________

________________________________________________________________

Study 2

Please select the most appropriate response for each of the following items.

Most of the time I feel that people...

|  | 1 (Disagree strongly) (1) | 2 (Disagree a little) (2) | 3 (Neither agree nor disagree) (3) | 4 (Agree a little) (4) | 5 (Agree strongly) (5) |
| --- | --- | --- | --- | --- | --- |
| ... like me as a person (1) |  |  |  |  |  |
| ... feel warmly toward me (2) |  |  |  |  |  |
| ... consider me to be a nice person to have around (3) |  |  |  |  |  |
| ... don't like me (4) |  |  |  |  |  |
| ... include me in their social activities (5) |  |  |  |  |  |
| ... are happy for me to belong to their social groups (6) |  |  |  |  |  |
| ... accept me (7) |  |  |  |  |  |
| ... see me as fitting in (8) |  |  |  |  |  |
| ... would be willing to be friends with me (9) |  |  |  |  |  |

Please select the most appropriate response for each of the following items.

Most of the time I feel that people...

|  | 1 (Disagree strongly) (1) | 2 (Disagree a little) (2) | 3 (Neither agree nor disagree) (3) | 4 (Agree a little) (4) | 5 (Agree strongly) (5) |
| --- | --- | --- | --- | --- | --- |
| ... respect my achievements (1) |  |  |  |  |  |
| ...value my opinions and ideas (2) |  |  |  |  |  |
| ...think highly of my abilities and talents (3) |  |  |  |  |  |
| ...admire me (4) |  |  |  |  |  |
| ...consider me a success (5) |  |  |  |  |  |
| ...look up to me (6) |  |  |  |  |  |
| ...see me as an important person (7) |  |  |  |  |  |
| ...consider me a high-status individual (8) |  |  |  |  |  |

Below is a list of statements dealing with your general feelings about yourself. Please respond using the following scale.

|  | Strongly Disagree 1 (1) | 2 (2) | 3 (3) | Neither Agree nor Disagree 4 (4) | 5 (5) | 6 (6) | Strongly Agree 7 (7) |
| --- | --- | --- | --- | --- | --- | --- | --- |
| On the whole, I am satisfied with myself. (1) |  |  |  |  |  |  |  |
| At times, I think I am no good at all. (2) |  |  |  |  |  |  |  |
| I feel that I have a number of good qualities. (3) |  |  |  |  |  |  |  |
| I am able to do things as well as most other people. (4) |  |  |  |  |  |  |  |
| I feel I do not have much to be proud of. (5) |  |  |  |  |  |  |  |
| I certainly feel useless at times. (6) |  |  |  |  |  |  |  |
| I feel that I’m a person of worth, at least on an equal plane with others. (7) |  |  |  |  |  |  |  |
| I wish I could have more respect for myself. (8) |  |  |  |  |  |  |  |
| All in all, I am inclined to feel that I am a failure. (9) |  |  |  |  |  |  |  |
| I take a positive attitude toward myself. (10) |  |  |  |  |  |  |  |

**Please indicate how much the following statements apply to you** using a response format ranging from "1 = not agree at all" to '6 = agree completely"

|  | 1 = Not agree at all (1) | 2 (4) | 3 (5) | 4 (6) | 5 (8) | 6 = Agree completely (9) |
| --- | --- | --- | --- | --- | --- | --- |
| I am great. (1) |  |  |  |  |  |  |
| I will someday be famous. (2) |  |  |  |  |  |  |
| I show others how special I am. (3) |  |  |  |  |  |  |
| I react annoyed if another person steals the show from me. (4) |  |  |  |  |  |  |
| I enjoy my successes very much. (5) |  |  |  |  |  |  |
| I secretly take pleasure in the failure of my rivals. (6) |  |  |  |  |  |  |
| Most of the time I am able to draw people's attention to myself in conversations. (7) |  |  |  |  |  |  |
| I deserve to be seen as a great personality. (8) |  |  |  |  |  |  |
| I want my rivals to fail. (9) |  |  |  |  |  |  |
| I enjoy it when another person is inferior to me. (10) |  |  |  |  |  |  |
| I often get annoyed when I am criticized. (11) |  |  |  |  |  |  |
| I can barely stand it if another person is at the center of events. (12) |  |  |  |  |  |  |
| Most people won't achieve anything. (13) |  |  |  |  |  |  |
| Other people are worth nothing. (14) |  |  |  |  |  |  |
| Being a very special person gives me a lot of strength. (15) |  |  |  |  |  |  |
| I manage to be the center of attention with my outstanding contributions. (16) |  |  |  |  |  |  |
| Most people are somehow losers. (17) |  |  |  |  |  |  |
| Mostly, I am very adept at dealing with other people. (18) |  |  |  |  |  |  |

Please answer the following questions by deciding to what extent each item is characteristic of your feelings and behavior. Fill in the blank next to each item by choosing a number from the scale provided below.

|  | Very uncharacteristic, strongly disagree (1) | Uncharacteristic (2) | Neutral (3) | Characteristic (4) | Very characteristic or true, strongly agree (5) |
| --- | --- | --- | --- | --- | --- |
| I can become entirely absorbed in thinking about my personal affairs, my health, my cares or my relations to others. (1) |  |  |  |  |  |
| My feelings are easily hurt by ridicule or the slighting remarks of others. (2) |  |  |  |  |  |
| When I enter a room I often become self-conscious and feel that the eyes of others are upon me. (3) |  |  |  |  |  |
| I dislike sharing the credit of an achievement with others. (4) |  |  |  |  |  |
| I feel that I have enough on my hands without worrying about other people's troubles. (5) |  |  |  |  |  |
| I feel that I am temperamentally different from most other people. (6) |  |  |  |  |  |
| I often interpret the remarks of others in a personal way. (7) |  |  |  |  |  |
| I easily become wrapped up in my own interests and forget the existence of others. (8) |  |  |  |  |  |
| I dislike being with a group unless I know that I am appreciated by at least one of those present. (9) |  |  |  |  |  |
| I am secretly "put out" or annoyed when other people come to me with their troubles, asking me for my time and sympathy. (10) |  |  |  |  |  |

Please select the appropriate response for each of the following items.

|  | 1 (Disagree strongly) (1) | 2 (Disagree a little) (2) | 3 (Neither agree nor disagree) (3) | 4 (Agree a little) (4) | 5 (Agree strongly) (5) |
| --- | --- | --- | --- | --- | --- |
| Above all, I want to be successful. (1) |  |  |  |  |  |
| Having the respect of others is essential to me. (2) |  |  |  |  |  |
| Being admired for my talents, and recognized for achievements, means everything to me. (3) |  |  |  |  |  |
| I aspire, first and foremost, to be a person of importance and distinction. (4) |  |  |  |  |  |
| Getting ahead of the competition really matters to me. (5) |  |  |  |  |  |
| Just being average really bothers me. (6) |  |  |  |  |  |
| I hate it when other people don't acknowledge my potential. (7) |  |  |  |  |  |
| I fear being insignificant, or being just another face in the crowd. (8) |  |  |  |  |  |
| Being disrespected by others is the worst thing in the world. (9) |  |  |  |  |  |
| I dread nothing more than being left behind in the struggle to survive. (10) |  |  |  |  |  |

Please select the most appropriate response for each of the following items.

|  | 1 (Strongly disagree) (1) | 2 (Disagree a little) (2) | 3 (Neither agree nor disagree) (3) | 4 (Agree a little) (4) | 5 (Agree strongly) (5) |
| --- | --- | --- | --- | --- | --- |
| Above all, I want to be accepted. (1) |  |  |  |  |  |
| Being liked by others is essential to me. (2) |  |  |  |  |  |
| Belonging to social groups, and being included in their activities, means everything to me. (3) |  |  |  |  |  |
| I desire, first and foremost, to have many friends and close relationships. (4) |  |  |  |  |  |
| Fitting in with others really matters to me. (5) |  |  |  |  |  |
| Even mild rejection really bothers me. (6) |  |  |  |  |  |
| I hate it when other people don't warm up to me. (7) |  |  |  |  |  |
| I fear not fitting in, or being someone others are indifferent to. (8) |  |  |  |  |  |
| Being disliked by others is the worst thing in the world. (9) |  |  |  |  |  |
| I dread nothing more than people excluding me from their social circle. (10) |  |  |  |  |  |

On this page you will find 60 items. Each item is scored on a **1 to 5 scale**, where 1 = the statement is false or that you **strongly disagree**; 2 = the statement is mostly false or you **disagree**; 3 = the statement is about equally true or false, you cannot decide, or you are **neutral** on the statement; 4 = the statement is mostly true or you **agree**; and 5 = the statement is definitely true or you **strongly agree**. Please read each item carefully and provide your answer that best corresponds to your agreement or disagreement. There are no right or wrong answers. Describe yourself honestly and state your opinions as accurately as possible.

|  | Disagree strongly (1) | Disagree a little (2) | Neither agree nor disagree (3) | Agree a little (4) | Agree strongly (5) |
| --- | --- | --- | --- | --- | --- |
| I am extremely ambitious. (1) |  |  |  |  |  |
| Others say I brag too much, but everything I say is true. (2) |  |  |  |  |  |
| Leadership comes easy for me. (3) |  |  |  |  |  |
| When someone does something nice for me, I wonder what they want from me. (4) |  |  |  |  |  |
| I deserve to receive special treatment. (5) |  |  |  |  |  |
| I get lots of enjoyment from entertaining others. (6) |  |  |  |  |  |
| It's fine to take advantage of persons to get ahead. (7) |  |  |  |  |  |
| I often fantasize about someday being famous. (8) |  |  |  |  |  |
| When people judge me, I just don't care. (9) |  |  |  |  |  |
| I don't worry about others' needs. (10) |  |  |  |  |  |
| I'm pretty good at manipulating people. (11) |  |  |  |  |  |
| I often feel as if I need compliments from others in order to be sure of myself. (12) |  |  |  |  |  |
| I hate being criticized so much that I can't control my temper when it happens. (13) |  |  |  |  |  |
| When I realize I have failed at something, I feel humiliated. (14) |  |  |  |  |  |
| I will try almost anything to get my "thrills." (15) |  |  |  |  |  |
| I have a tremendous drive to succeed. (16) |  |  |  |  |  |
| I only associate with people of my caliber. (17) |  |  |  |  |  |
| I am comfortable taking on positions of authority. (18) |  |  |  |  |  |
| I trust that other people will be honest with me. (19) |  |  |  |  |  |
| I don't think the rules apply to me as much as they apply to others. (20) |  |  |  |  |  |
| I like being noticed by others. (21) |  |  |  |  |  |
| I will use persons as tools to advance myself. (22) |  |  |  |  |  |
| I often fantasize about having lots of success and power. (23) |  |  |  |  |  |
| I don't really care what others think of me. (24) |  |  |  |  |  |
| I don't generally pay attention to the woes of others. (25) |  |  |  |  |  |
| I can maneuver people into doing things. (26) |  |  |  |  |  |
| I am stable in my sense of self. (27) |  |  |  |  |  |
| I have at times gone into a rage when not treated rightly. (28) |  |  |  |  |  |
| I feel awful when I get put down in front of others. (29) |  |  |  |  |  |
| I am a bit of a daredevil. (30) |  |  |  |  |  |
| I aspire for greatness. (31) |  |  |  |  |  |
| I do note waste my time hanging out with people who are beneath me. (32) |  |  |  |  |  |
| Persons generally follow my lead and authority. (33) |  |  |  |  |  |
| I'm slow to trust people. (34) |  |  |  |  |  |
| It may seem unfair, but I deserve extra (i.e., attention, privilieges, rewards). (35) |  |  |  |  |  |
| I like being the most popular person at a party. (36) |  |  |  |  |  |
| Sometimes to succeed you need to use other people. (37) |  |  |  |  |  |
| I rarely fantasize about becoming famously successful. (38) |  |  |  |  |  |
| I'm pretty indifferent to the criticism of others. (39) |  |  |  |  |  |
| I'm not big on feelings of sympathy. (40) |  |  |  |  |  |
| I can talk my way into and out of anything. (41) |  |  |  |  |  |
| I feel very insecure about whether I will achieve much in life. (42) |  |  |  |  |  |
| It really makes me angry when I don't get what I deserve. (43) |  |  |  |  |  |
| I feel ashamed when people judge me. (44) |  |  |  |  |  |
| I would risk injury to do something exciting. (45) |  |  |  |  |  |
| I am driven to succeed. (46) |  |  |  |  |  |
| I am a superior person. (47) |  |  |  |  |  |
| I tend to take charge of most situations. (48) |  |  |  |  |  |
| I often think that others aren't telling me the whole truth. (49) |  |  |  |  |  |
| I believe I am entitled to special accommodations. (50) |  |  |  |  |  |
| I love to entertain people. (51) |  |  |  |  |  |
| I'm willing to exploit others to further my own goals. (52) |  |  |  |  |  |
| Someday I believe that most people will know my name. (53) |  |  |  |  |  |
| Others' opinions of me are of little concern to me. (54) |  |  |  |  |  |
| I don't get upset by the suffering of others. (55) |  |  |  |  |  |
| It is easy to get people to do what I want. (56) |  |  |  |  |  |
| I wish I didn't care so much about what others think of me. (57) |  |  |  |  |  |
| I feel enraged when people disrespect me. (58) |  |  |  |  |  |
| I feel foolish when I make a mistake in front of others. (59) |  |  |  |  |  |
| I like doing things that are risky or dangerous. (60) |  |  |  |  |  |

People have all kinds of private thoughts about themselves. From person to person, these self-thoughts can vary quite a lot in content. We are interested in the sort of self-thoughts you possess. Below you will find a list of self-thoughts you may have. For each self-thought, please indicate whether you have this or a similar thought. Be as honest as possible. remember, your responses are totally anonymous.

|  | 1 = Disagree strongly (1) | 2 (2) | 3 (3) | 4 (4) | 5 (5) | 6 (6) | 7 = Agree strongly (7) |
| --- | --- | --- | --- | --- | --- | --- | --- |
| I am the most helpful person I know. (1) |  |  |  |  |  |  |  |
| I am going to bring peace to the world. (2) |  |  |  |  |  |  |  |
| I am the best friend someone can have. (3) |  |  |  |  |  |  |  |
| I will be well known for the good deeds I will have done. (4) |  |  |  |  |  |  |  |
| I am (going to be) the best parent on this planet. (5) |  |  |  |  |  |  |  |
| I am the most caring person in my social surrounding. (6) |  |  |  |  |  |  |  |
| In the future I will be well known for solving the world's problems. (7) |  |  |  |  |  |  |  |
| I greatly enrich others' lives. (8) |  |  |  |  |  |  |  |
| I will bring freedom to the people. (9) |  |  |  |  |  |  |  |
| I am an amazing listener. (10) |  |  |  |  |  |  |  |
| I will be able to solve world poverty. (11) |  |  |  |  |  |  |  |
| I have a very positive influence on others. (12) |  |  |  |  |  |  |  |
| I am generally the most understanding person. (13) |  |  |  |  |  |  |  |
| I'll make the world a much more beautiful place. (14) |  |  |  |  |  |  |  |
| I am extraordinarily trustworthy. (15) |  |  |  |  |  |  |  |
| I will be famous for increasing people's well-being. (16) |  |  |  |  |  |  |  |

Please fill in the demographic questions below. This information is helpful to ensure that we have a representative sample of participants in our study.

Age:

________________________________________________________________

Gender (select one):

- Male (1)
- Female (2)
- Other (3)
- Rather not specify (4)

Which of the following BEST describes your background?

- Aboriginal/First Nations/Metis (1)
- White/European (2)
- Black/African/Caribbean (3)
- Southeast Asian (e.g., Chinese, Japanese, Korean, Vietnamese, Cambodian, Filipino, etc.) (4)
- Arab (Saudi Arabian, Palestinian, Iraqi, etc.) (5)
- South Asian (East Indian, Sri Lankan, etc.) (6)
- Latin American (Costa Rican, Guatemalan, Brazilian, Columbian, etc.) (7)
- West Asian (Iranian, Afghani, etc.) (8)
- Other (9)

How good is your English?

- Excellent (1)
- Good (2)
- Average (3)
- Poor (4)
- Very Poor (5)

What do you think this study was about?

________________________________________________________________

When conducting research, we rely on participants' responses being honest and accurate in order for us to draw valid conclusions from the data. However, we recognize that there are many reasons participants might be unable or unwilling to provide fully honest and accurate responses. In these cases it is truly helpful for us to be able to identify responses that may not be valid so we can take this into account.
 
In your honest opinion, should we use your data from this survey? 
  
(Please note your answer is **confidential and you will be compensated** whichever answer you choose.)

- Yes (1)
- No (2)

If you selected "No", please specify: Why do you think we should **NOT** use your data?

________________________________________________________________

________________________________________________________________

________________________________________________________________

________________________________________________________________
